# Supplementary material for: High-purity ethylene production via indirect carbon dioxide electrochemical reduction
Source: Nat Commun. 2024 Jul 19;15:6078. doi: 10.1038/s41467-024-50522-7 (PMC11271605; doi:10.1038/s41467-024-50522-7)
Supplement: Supplementary file 1 — Supplementary Information [file 41467_2024_50522_MOESM1_ESM.pdf]

High-purity Ethylene Production via Indirect Carbon Dioxide Electrochemical Reduction

Wenpeng Ni, Houjun Chen, Naizhuo Tang, Ting Hu, Wei Zhang, Yan Zhang, and Shiguo Zhang*

College of Materials Science and Engineering, Hunan University, Changsha 410004, China

E-mail: zhangsg@hnu.edu.cn

Table of Contents

Supplementary figures

Supplementary tables

Supplementary references

Supplementary figures

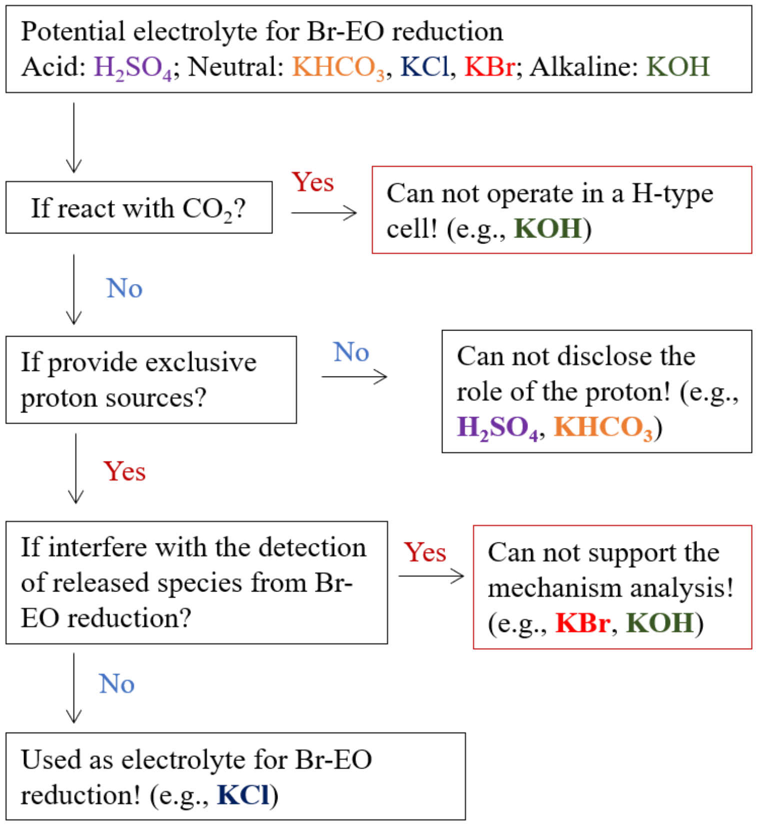

Fig. S1 The selection route for the electrolyte.

For the electrochemical assessment and mechanism analysis of Br-EO reduction, we adopted 0.5 M KCl aqueous solution as the electrolyte, mainly based on the following considerations. A) To simulate the electrolyte after CO_2RR, we considered alkaline (e.g., KOH), neutral (e.g., $KHCO_3$, KCl), and acidic electrolyte (H_2SO_4) as the candidates. However, only neutral electrolytes align with the research objectives of this work. As for alkaline electrolytes, CO_2 would react with OH^- in the bulk of electrolytes, given the utilization of an H-type cell. Concerning acidic electrolytes, there are two potential proton sources, H^+ and H_2O molecules. It is better to utilize electrolytes with exclusive proton sources to simplify the mechanism exploration. In this context, a neutral electrolyte, excluding $KHCO_3$ (HCO_3^- also serves as a proton donor), emerges as the optimal choice for our inquiry. B) The reduction of Br-EO involved the removal of the -Br and -OH groups. Discerning the resultant species is pivotal to unraveling the underlying reaction mechanism, necessitating the elimination of bromide and hydroxide species from the electrolyte to preclude interference. Hence, from a mechanistic standpoint, we employed a 0.5 M KCl electrolyte for Br-EO reduction. But in the integrated CO_2-to-Br-EO and Br-EO-to-ethylene system, the KBr was utilized as the anolyte for the first electrolytic cell.

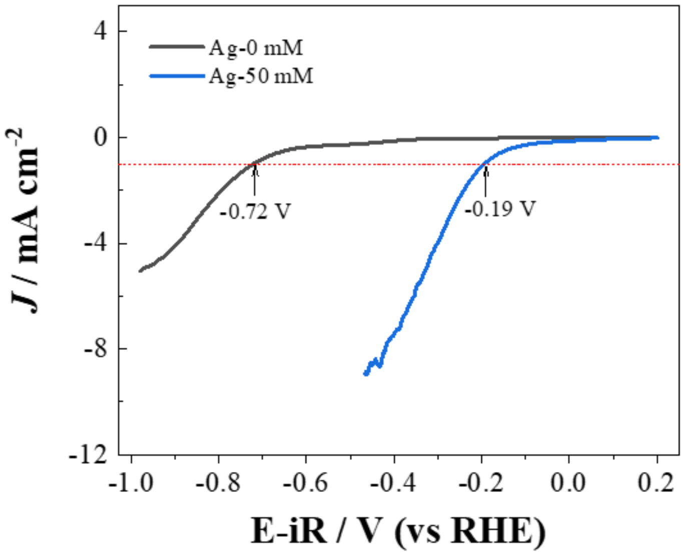

Fig. S2 LSV curves of Ag-foil electrode in 0.5 M KCl with and without 50 mM Br-EO, under CO_2 bubbling (30 sccm). The scan rate is 10 mV s^{-1}.

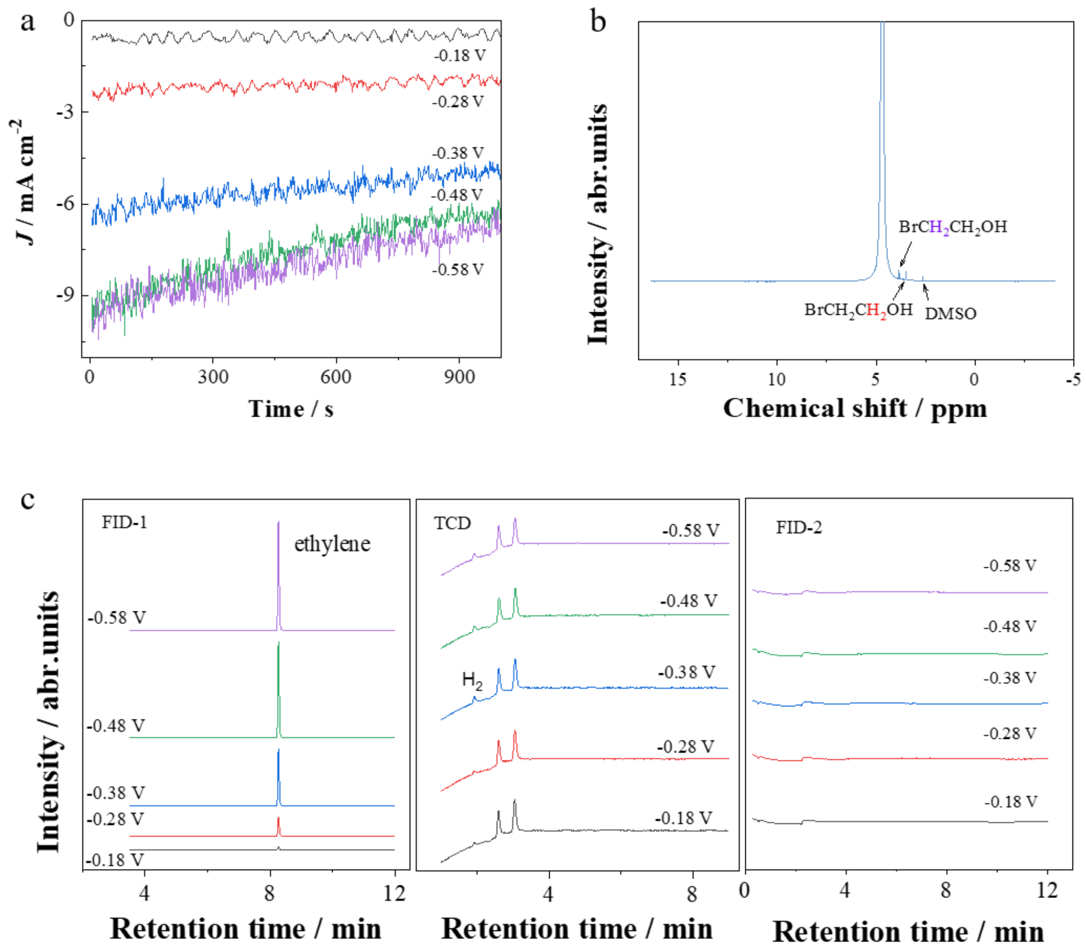

Fig. S3 Product analysis for Br-EO reduction. (a) I-t curves for pristine Ag-foil electrode in 0.5 M KCl containing 50 mM Br-EO, under CO_2 bubbling (30 sccm). The potential is iR compensate. (b) ^{1}H NMR of the electrolyte after electrolysis at -0.18 V over Ag foil electrode. (c) Online GC spectra at different potentials over the detectors of FID-1, TCD, and FID-2.

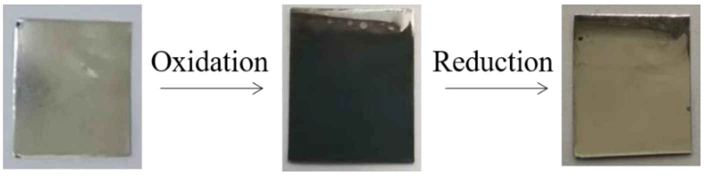

Fig. S4 The digital photos of Ag-foil electrode after oxidation and subsequent reduction step.

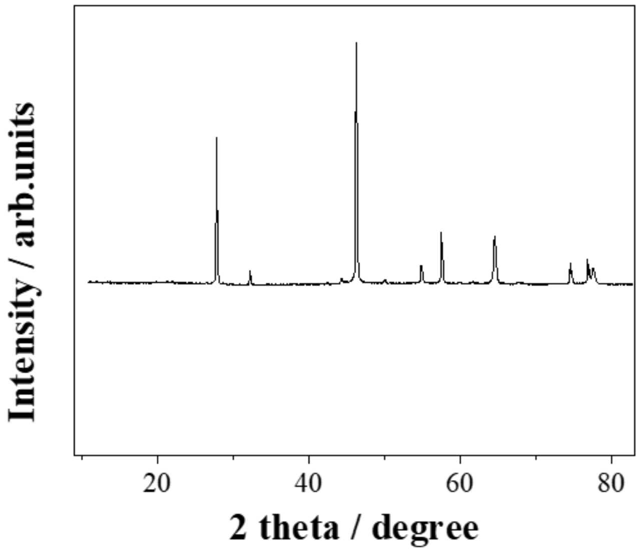

Fig. S5 The XRD pattern of Ag foil electrode after oxidation in 0.5 M KCl electrolyte.

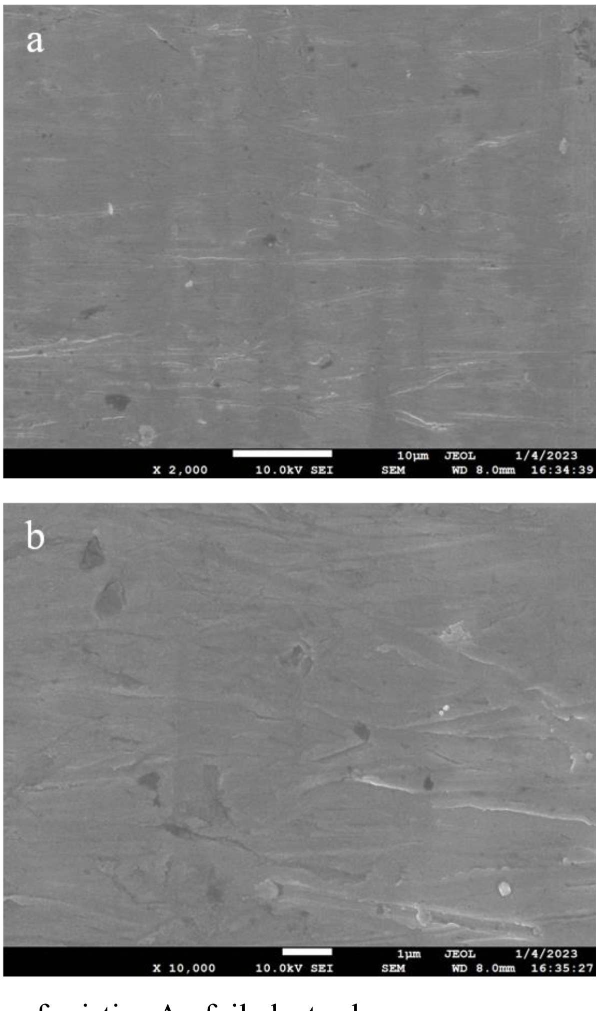

Fig. S6 SEM images of pristine Ag foil electrode.

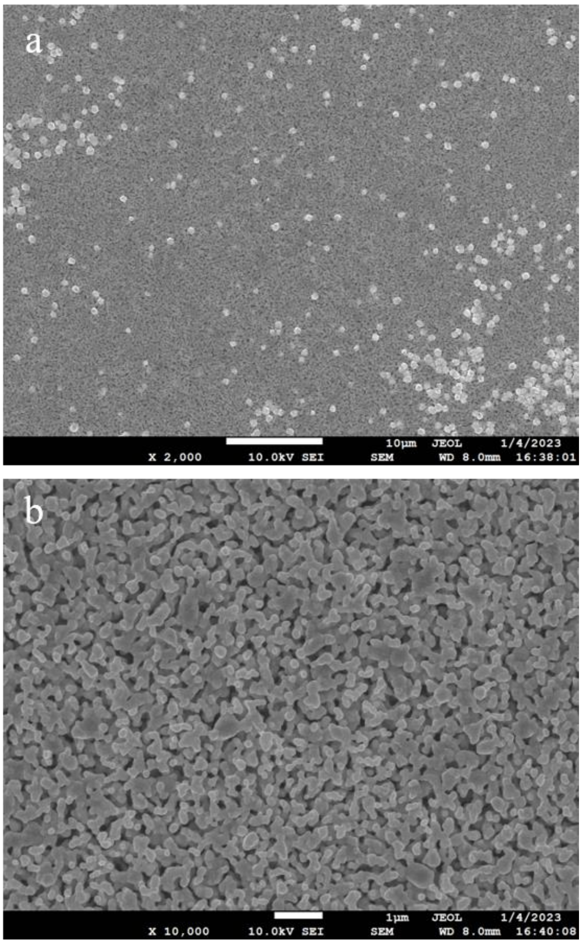

Fig. S7 SEM images of AC-Ag.

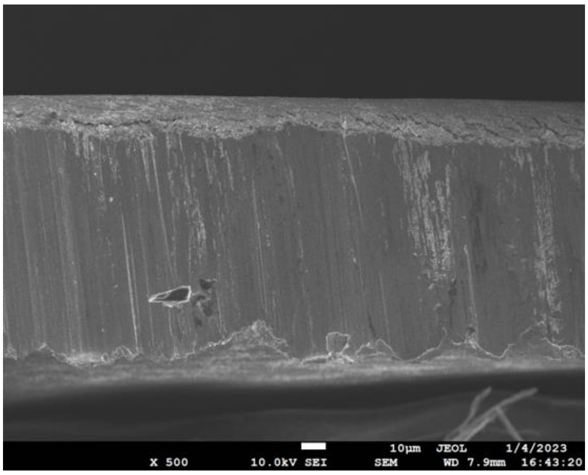

Fig. S8 Cross-sectional SEM image of AC-Ag.

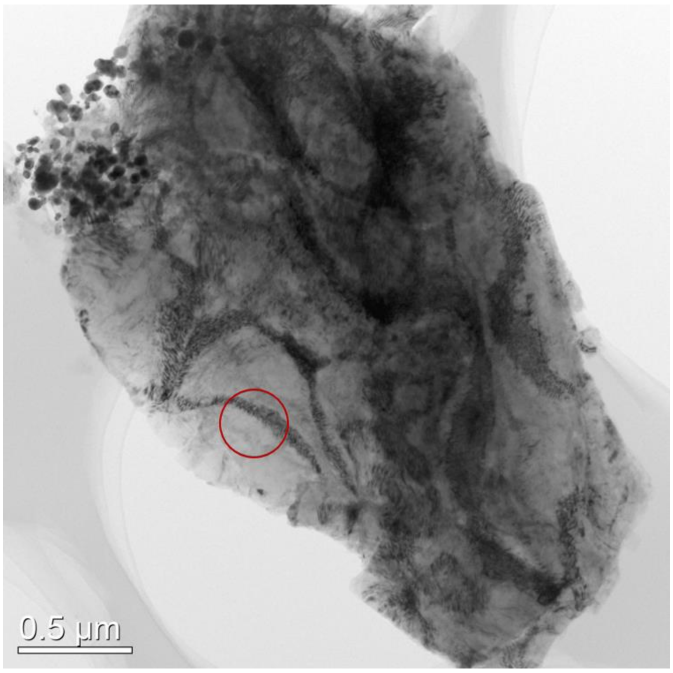

Fig. S9 Area in TEM image for SEAD measurement of AC-Ag.

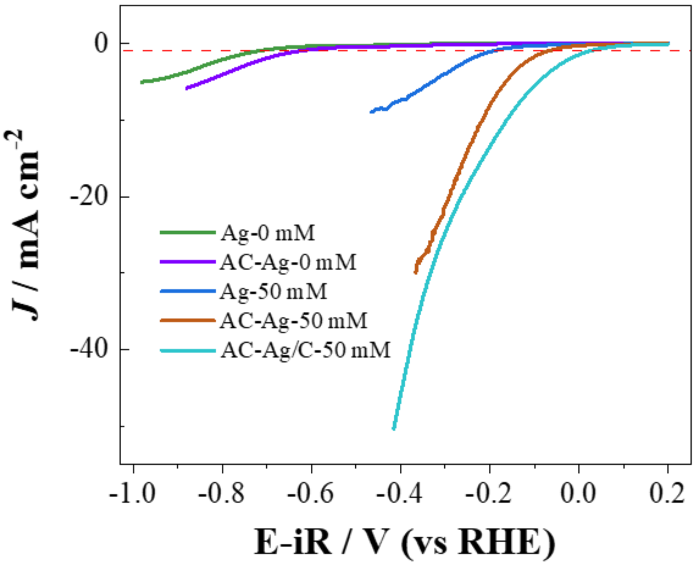

Fig. S10 LSV curves for Br-EO reduction over different Ag-based catalysts in CO_2-saturated 0.5 M KCl aqueous solution. Scan rate is 10 mV s^{-1}. The size of Ag and AC-Ag are both 1 cm^2. The mass loading for AC-Ag/C is 1 mg cm^{-2}.

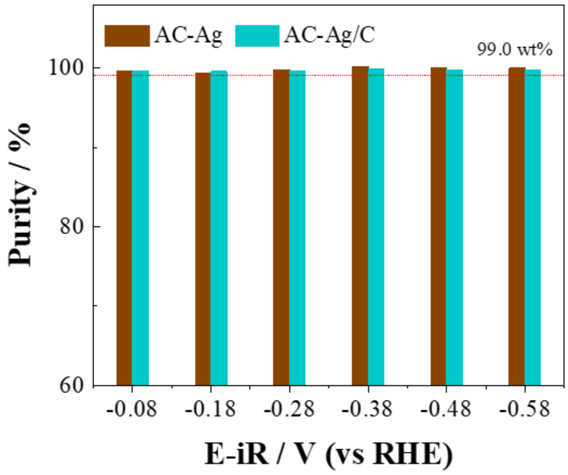

Fig. S11 The purity of ethylene over AC-Ag and AC-Ag/C at different potentials in 0.5 M KCl with 50 mM Br-EO.

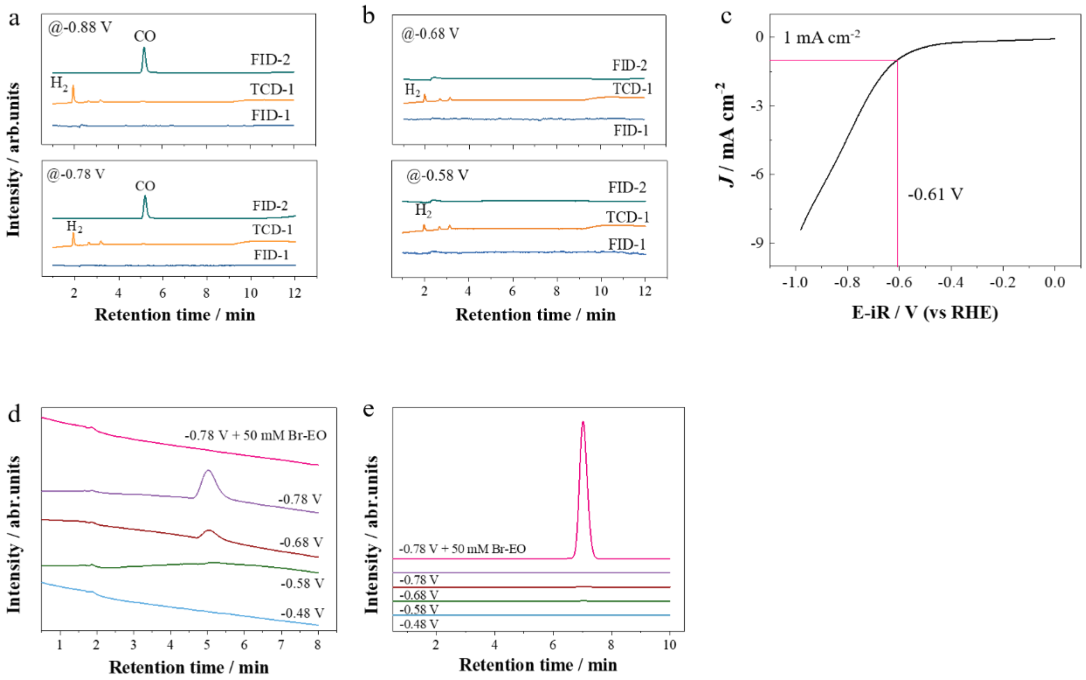

Fig. S12 Product characterizations. GC spectra of (a) pristine Ag electrode at -0.78 and -0.88 V (vs RHE) in 0.5 M KCl. (b) AC-Ag electrode at -0.58 and -0.68 V (vs RHE) in 0.5 M KCl. (c) LSV curve of AC-Ag/C catalyst in 0.5 M KCl saturated with CO_2 (30 sccm). GC spectra of (d) FID for detecting CO, (e) FID for detecting ethylene of AC-Ag/C catalyst in 0.5 M KCl at different potentials and in 0.5 M KCl containing 50 mM Br-EO at -0.78 V (vs RHE). All potentials are IR compensated.

Supplementary Note 1

Herein, Ag was chosen as the catalyst for the reduction of Br-EO because its remarkable catalytic ability in cleaving the C-halogen bond. On the other hand, Ag-based catalysts, such as Ag nanoplates, nanowires, or single-atom, have also shown significant efficacy in CO_2-to-CO conversion. But in our present investigation, no discernible formation of CO was observed via online GC analysis during the reduction of Br-EO. Several factors may account for this result.

Firstly, the potential window examined for the reduction of Br-EO (-0.08 ~ -0.58 V, vs RHE) does not encompass the reduction bias necessary for the generation of detectable concentrations of CO. We measured the reduction product distribution over Ag and AC-Ag electrode for pure CO_2 reduction. As shown in Fig. S12a, the Ag foil electrode gave a detectable CO peak at the potentials of -0.78 and -0.88 V (vs RHE, the current density was insufficient to yield adequate products for GC analysis at more positive potentials). While for AC-Ag, only H_2 was observed at -0.58 and -0.68 V (vs RHE, Fig. S12b). These results align with previously reported initial reduction potentials of Ag foil electrodes for CO_2 reduction. To evaluate the activity of the AC-Ag/C catalyst for CO production, the LSV curve in CO_2-saturated 0.5 M KCl was collected. It is evident that the initial potential, defined by the potential at which the current reaches 1 mA cm^{-2}, stands at -0.61 V (vs RHE, Fig. S12c). On-line GC analysis was conducted from -0.48 to -0.78 V (vs RHE), encompassing the applied potential for Br-EO reduction (-0.48 and -0.58 V, vs RHE). However, the appearance of the CO peak was not observed until the potential reached -0.68 V (vs RHE), suggesting that CO_2 reduction over the catalysts employed in our study occurs at a potential outside the range of focus for the Br-EO reduction in this work.

Secondly, the presence of Br-EO in electrolytes will lead to the competitive adsorption between Br-EO and CO_2, thereby suppressing the CO_2 electroreduction. At the potential of -0.78 V (vs RHE), wherein CO can be discerned in CO_2-saturated 0.5 M KCl, the CO peak disappeared upon the introduction of 50 mM Br-EO into the electrolyte (Fig. S12d). Instead, a pronounced peak denoting the presence of C_2H_4 emerges (Fig. S12e). Detailed analysis through in situ ATR-SEIRAS revealed that all traces of CO_2 reduction intermediates disappeared, leaving only signals indicative of Br-EO reduction, in 0.5 M KCl with 50 mM Br-EO. These results collectively demonstrated that Br-EO in electrolytes exhibits a marked predilection for reduction, thereby implying a paucity of active sites available for the adsorption and subsequent

reduction of CO_2.

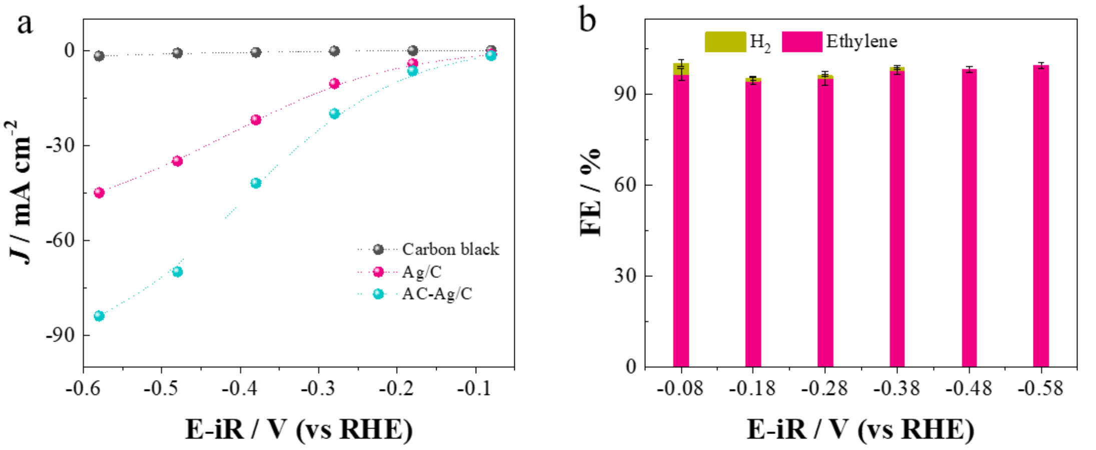

Fig. S13 Electrochemical data for Ag/C electrode. (a) The J of carbon black, Ag/C, and AC-Ag/C for Br-EO reduction (50 mM) in 0.5 M KCl aqueous solution. (b) The Faradaic efficiency of H_2 and ethylene for Ag/C. Error bars correspond to the standard deviation of three measurements.

Supplementary Note 2

We assessed the electrochemical performance of the Br-EO reduction over pristine carbon black and Ag/C without electrochemical activation (denoted as Ag/C), in 0.5 M KCl while CO_2 bubbled through. Within the potential range like AC-Ag/C (-0.18 ~ -0.58 V vs RHE), the pristine carbon black showed an almost negligible current response, exemplified by J registering at only -1.8 mA cm^{-2} at -0.58 V (vs RHE), which is significantly inferior to that of AC-Ag/C (Fig. S13a). Noteworthy is that ethylene production was only discerned at -0.48 and -0.58 V (vs RHE), with selectivity of 50.99% and 74.4%. Regarding the Ag/C sample, it demonstrated a significantly enhanced reductive current, boasting a J of -45.0 mA cm^{-2} at -0.58 V (vs RHE). A selectivity analysis revealed the near-exclusive production of ethylene, with only a marginal generation of H_2, manifesting faradaic efficiency lower than 4.0% at all potentials (Fig. S13b). However, despite the notable high selectivity for ethylene observed in the case of Ag/C, its current densities are still lower than that of AC-Ag/C. These results can conclude that the excellent activity of AC-Ag/C is attributable to the presence of Ag particles, and the electrochemical activation can further improve the activity for Br-EO reduction.

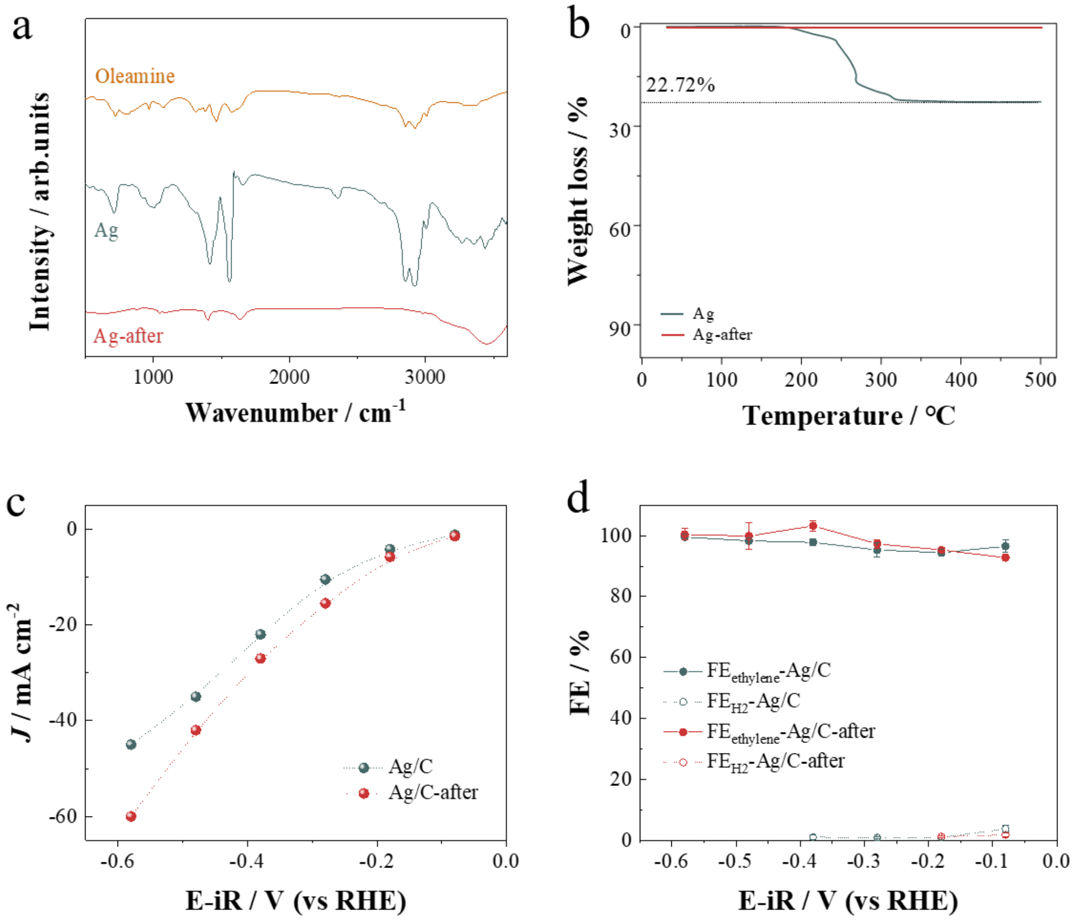

Fig. S14 Analysis for the potential influence of residue capping agent. (a) FTIR, (b) TGA curves of pristine Ag particle and Ag particle after thermal treatment. (c) Total current density, and (d) FE of H_2 and ethylene for Ag/C and Ag/C after thermal treatment in 0.5 M KCl containing 50 mM Br-EO with CO_2 bubbling (30 sccm). Error bars correspond to the standard deviation of three measurements.

Supplementary Note 3

Oleamine served as the capping agent to imbue stability upon the Ag nanoparticles. Nevertheless, these residual organic molecules may influence the electrochemical behavior of the as-obtained Ag nanoparticles. According to the FTIR analysis, our synthesized Ag particles, in their as-yielded state, exhibit vestiges of oleamine adorning their surfaces, evidenced by the characteristic absorption peaks corresponding to pure oleamine (Fig. S14a). The TGA curve of pristine Ag particles, conducted under an Ar atmosphere, commences a mass loss at approximately 200 °C, ultimately registering a weight reduction of 22.72% (Fig. S14b).

To scrutinize the influence of lingering oleamine molecules on the reduction of Br-

EO catalyzed by Ag/C, we subjected the Ag/C catalyst to treatment at 250 ^oC, hereinafter referred to as Ag/C-after. The removal of oleamine was substantiated by the disappearance of characteristic oleamine peaks in the FTIR spectrum, accompanied by an absence of mass loss in the TGA curve. To exclude the potential influence of electrochemical activation procedures on the residual surface oleamine molecule, we conducted a comparative analysis of the electrochemical performance between the pristine Ag/C and the Ag/C-after without electrochemical activation. In terms of current density, it was observed that Ag/C-after could deliver a slightly superior current when compared to the Ag/C counterpart (Fig. S14c), likely attributable to the enhanced exposure of active sites. However, regarding the selectivity for ethylene and H_2, both Ag/C and Ag/C-after exhibited comparable Faradaic efficiencies (Fig. S14d). These findings collectively substantiate that the pronounced selectivity for the conversion of Br-EO to ethylene primarily emanates from the Ag particles themselves, rather than the remnants of oleamine on their surfaces.

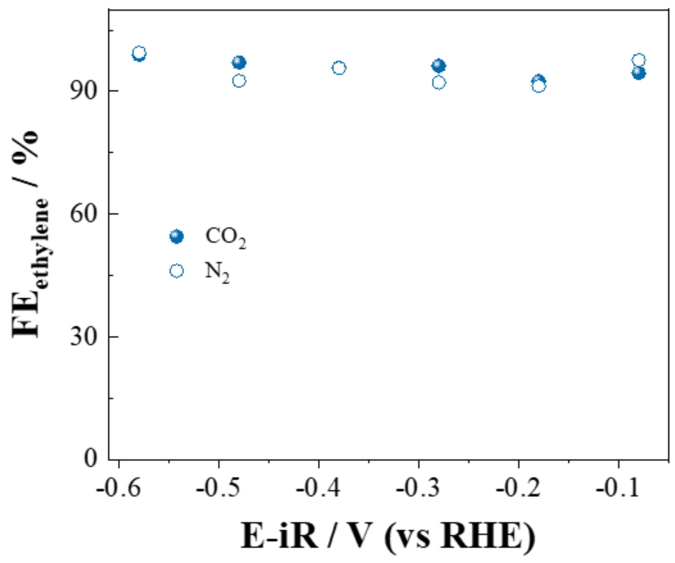

Fig. S15 FE$_{ethylene}$ for AC-Ag electrode in CO_2- and N_2-saturated 0.5 M KCl with the gas flow rate of 30 sccm.

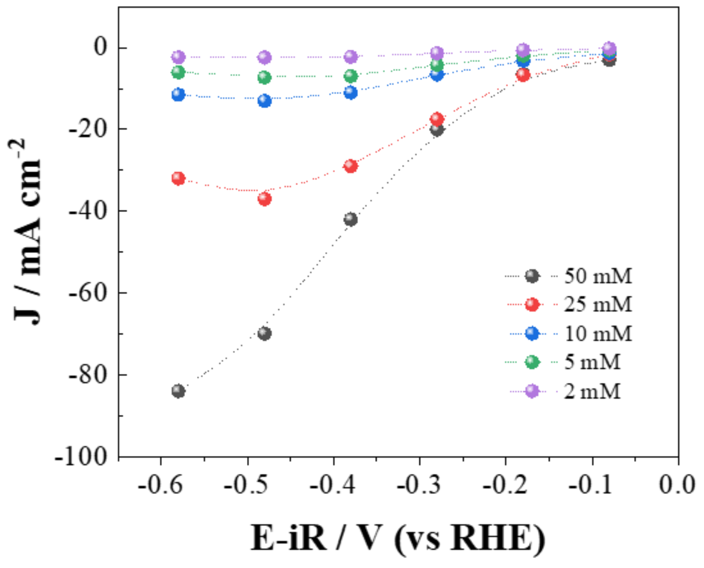

Fig. S16 Total current density of Br-EO reduction with different concentrations for AC-Ag/C electrode in 0.5 M KCl aqueous solution.

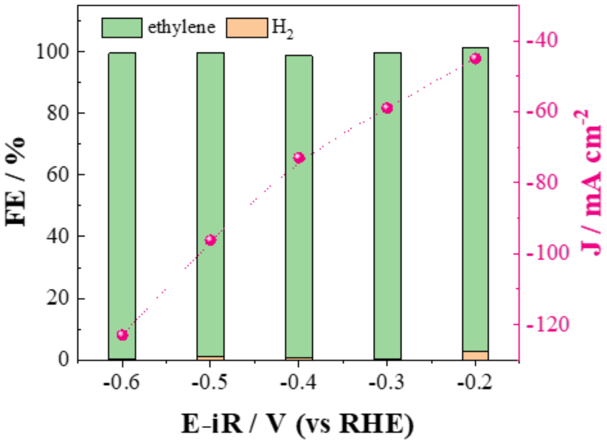

Fig. S17 FE of H_2 and ethylene, as well as the total current density, for the Br-EO reduction tested by a flow-through cell over the AC-Ag/C catalyst, using 0.5 M KCl electrolyte with 50 mM Br-EO.

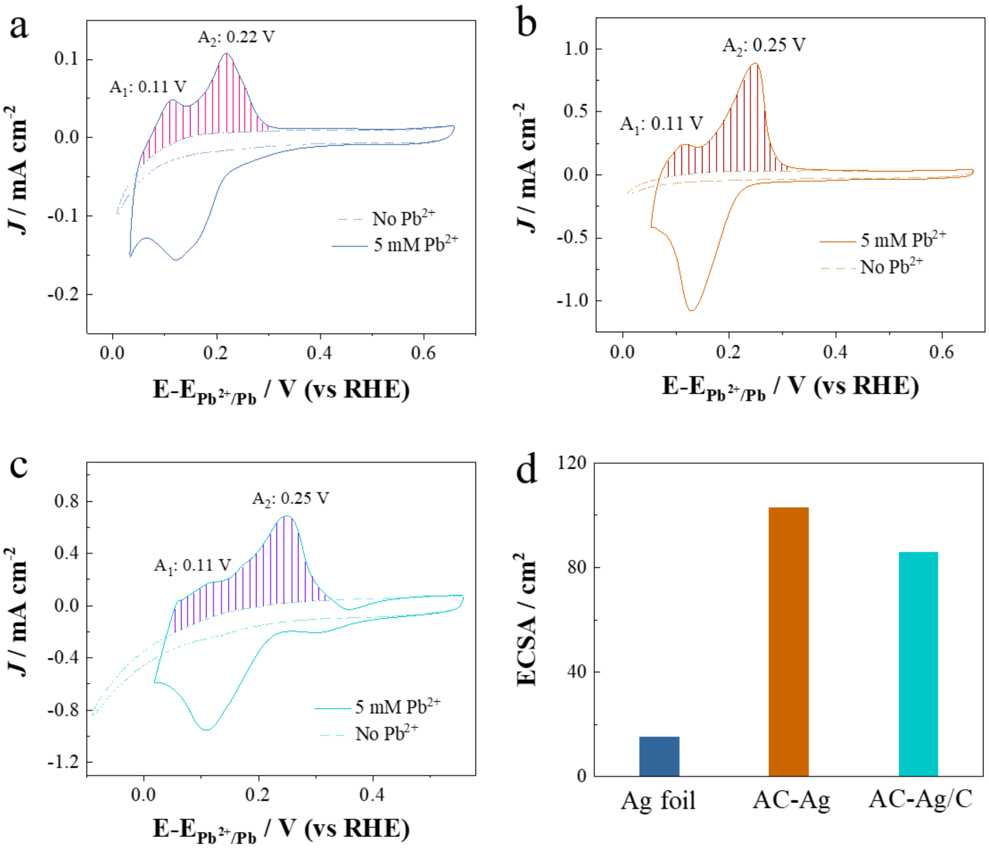

Fig. S18 Electrochemical active specific area analysis. Pb underpotential deposition of (a) Ag foil, (b) AC-Ag, and (c) AC-Ag/C. (d) Electrochemical active surface areas of different Ag electrodes.

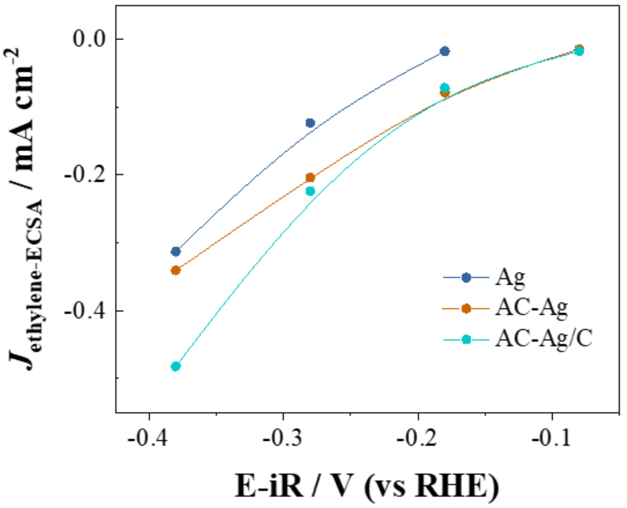

Fig. S19 ECSA normalized partial ethylene current density for Ag, AC-Ag, and AC-Ag/C electrodes.

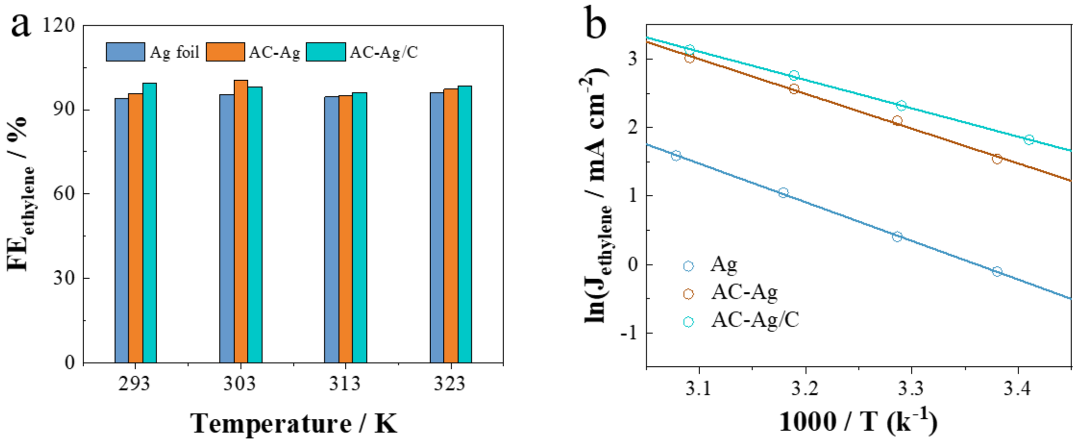

Fig. S20 Activation energy analysis. (a) $FE_{ethylene}$ of Ag foil, AC-Ag, and AC-Ag/C at -0.38 V (vs RHE) under different temperatures, in 0.5 M KCl with 50 mM Br-EO. The size for Ag foil and AC-Ag is 1 cm^2. The mass loading for AC-Ag/C is 1 mg cm^{-2}. (b) Linear fitting of the natural logarithm of the ethylene partial current densities versus the inverse temperatures.

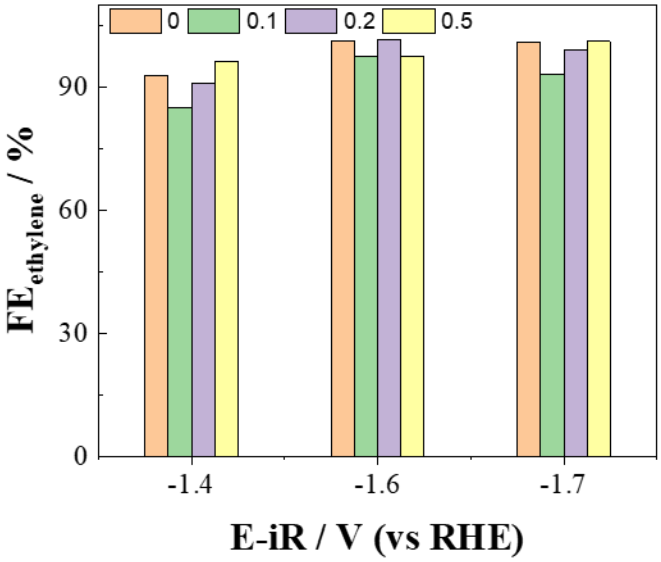

Fig. S21 Variations of $FE_{ethylene}$ with the changes of H_2O concentration in DMSO-based electrolyte over AC-Ag/C electrode, with the Br-EO concentration of 50 mM.

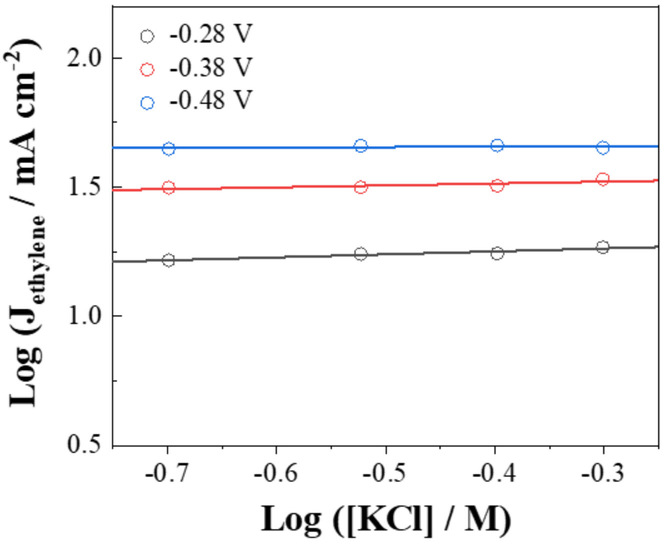

Fig. S22 Dependence of $J_{ethylene}$ and the concentration of KCl. The Br-EO concentration is 50 mM and the working electrode is AC-Ag/C with mass loading of 1 mg cm^{-2}. The potentials are iR compensated.

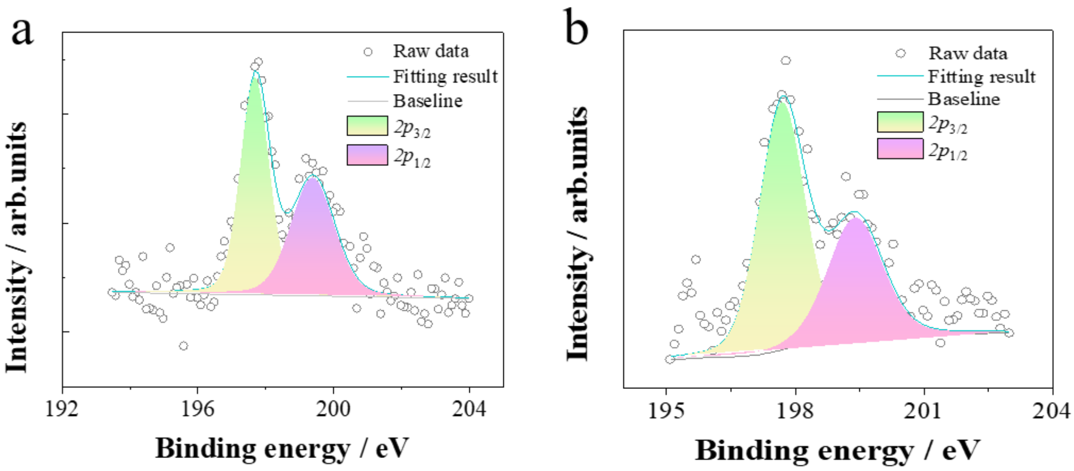

Fig. S23 Cl *2p* XPS spectra for AC-Ag/C after electrochemical testing at -0.58 V (vs RHE) for 2 h in (a) 0.5 M KCl, and (b) 0.1 M $KHCO_3$.

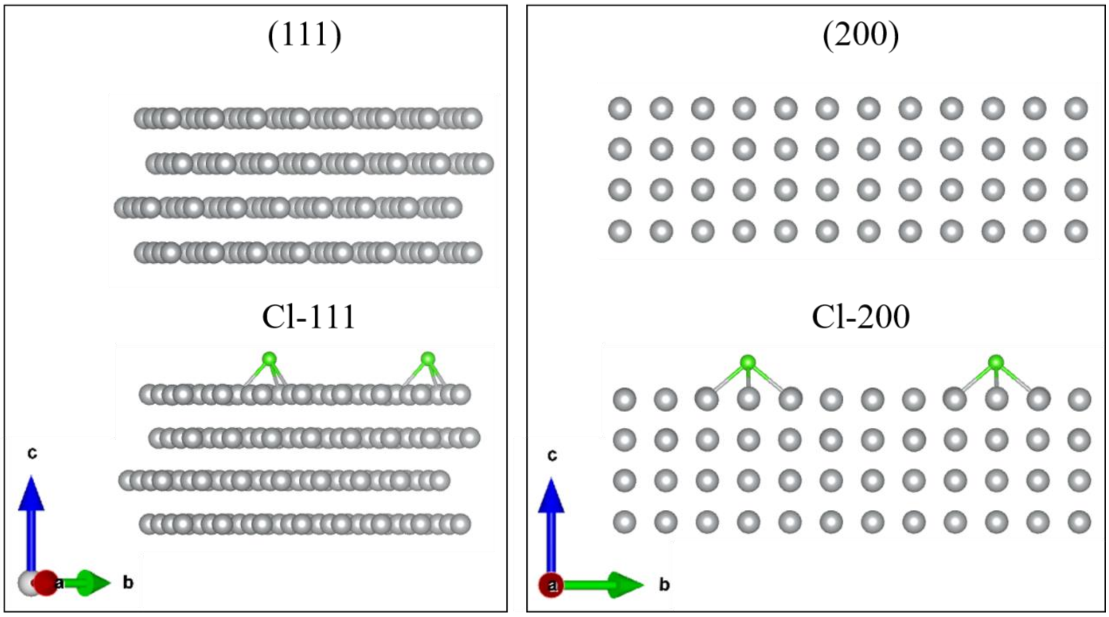

Fig. S24 Models for theoretical calculation. Ag (111) and (200) plane, as well as their corresponding Cl-incorporated counterparts (Cl-111 and Cl-200).

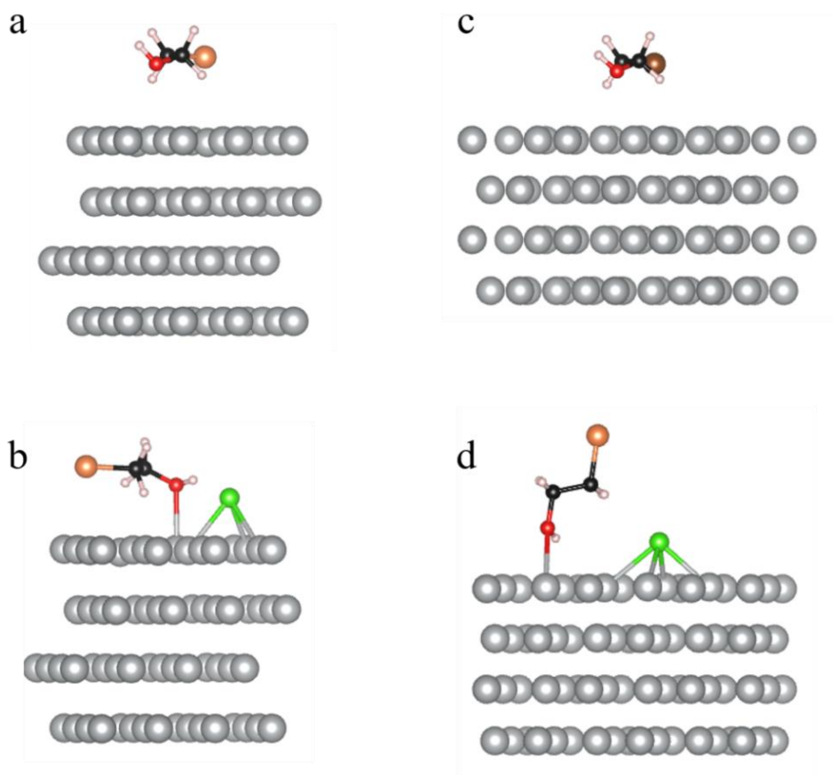

Fig. S25 Adsorption configurations of Br-CH_2CH_2-OH. (a) Ag (111), (b) Cl-111, (c) Ag (200), and (d) Cl-200.

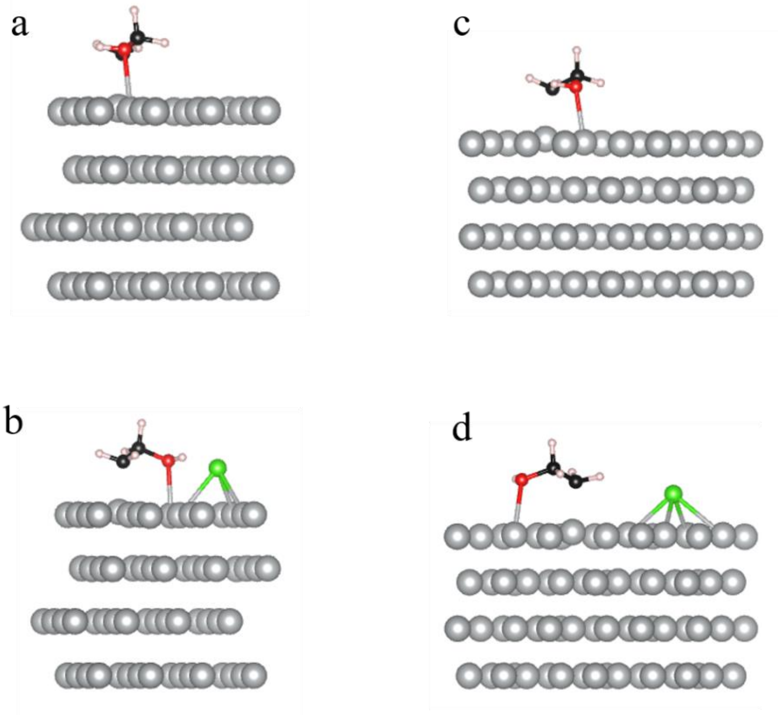

Fig. S26 Adsorption configurations of *CH_2CH_2-OH. (a) Ag (111), (b) Cl-111, (c) Ag (200), and (d) Cl-200.

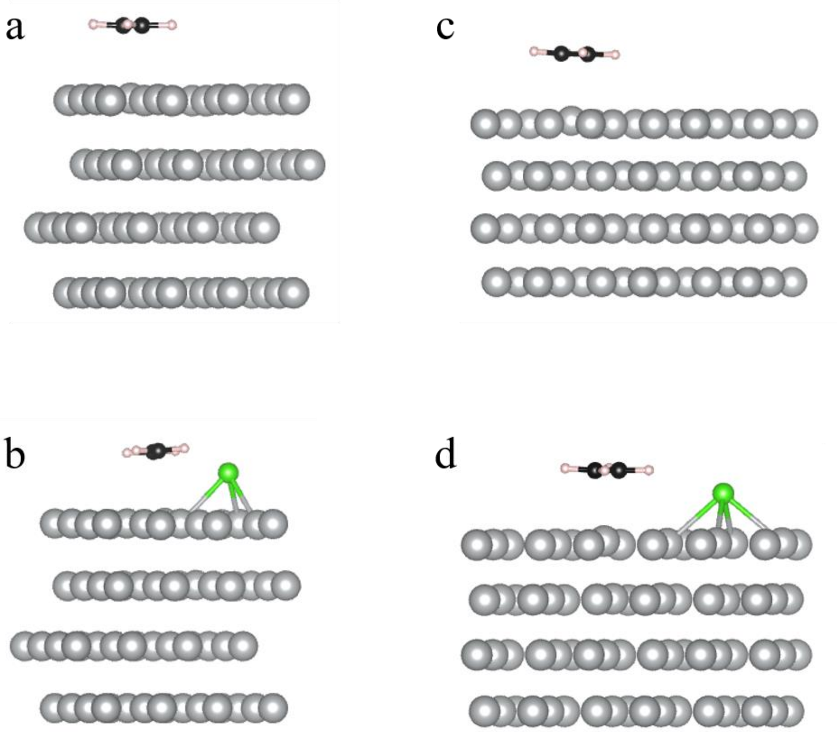

Fig. S27 Adsorption configurations of *CH_2CH_2*. (a) Ag (111), (b) Cl-111, (c) Ag (200), and (d) Cl-200.

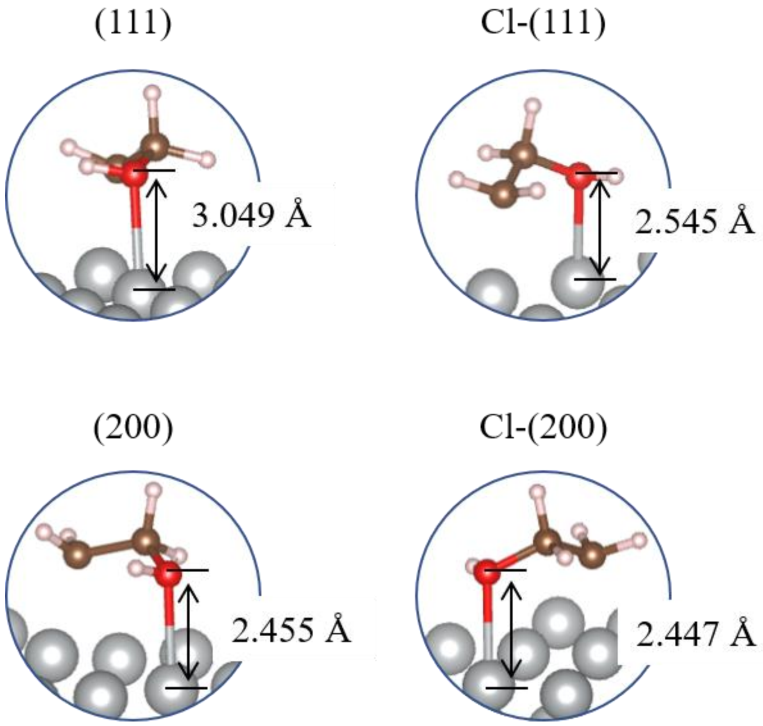

Fig. S28 The length of Ag-O bond for the adsorption of *$HOCH_2CH_2$ on Ag (111), Cl-(111), Ag (200), and Cl-(200) models.

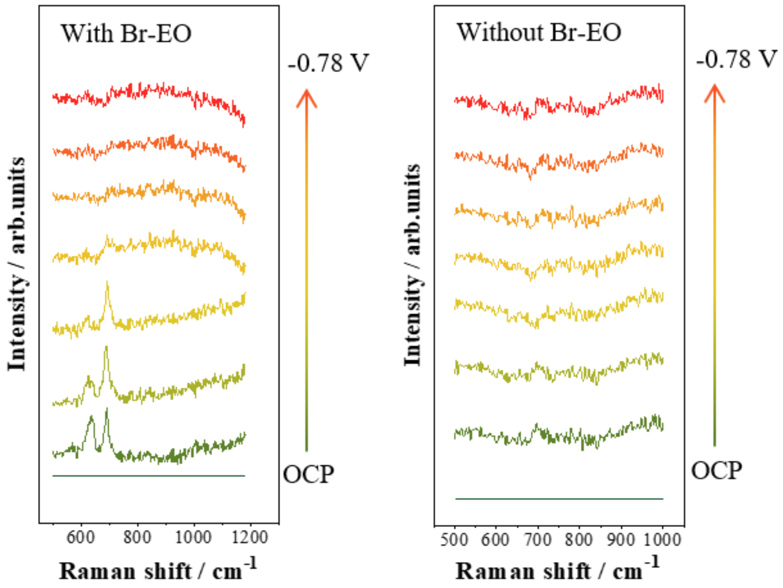

Fig. S29 In situ Raman spectra for AC-Ag/C catalysts in 0.5 M KCl electrolyte with and without 50 mM Br-EO, from open circuit potential (OCP) to -0.78 V (vs RHE).

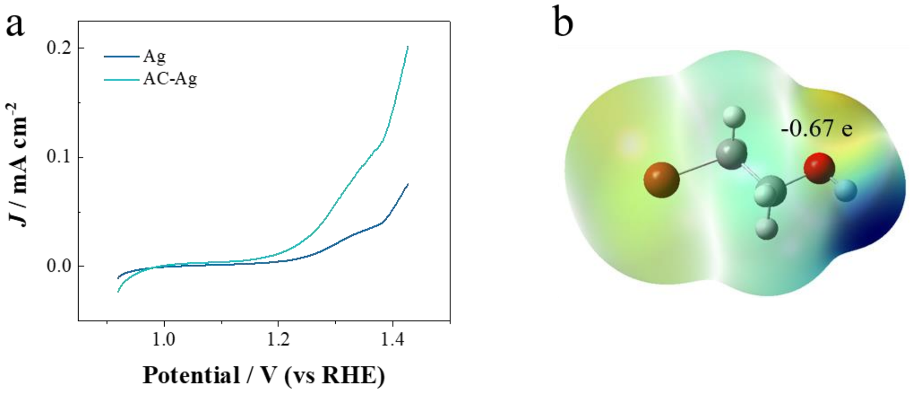

Fig. S30 Ag-O interaction analysis. (a) LSV curve for Ag and AC-Ag electrode collected in N_2-saturated 0.1 M KOH aqueous electrolyte. The scan rate is 10 mV s^{-1}. (b) The charge distribution over Br-EO molecule.

Supplementary Note 4

The adsorption of OH^- typically occurs via bonding with the adsorption site through the O atom. Thus, we collected the LSV curves for OH^- adsorption to demonstrate the higher oxygen affinity of the Cl-doped Ag site. The lower onset potential for the OH^- adsorption of AC-Ag compared to the pristine Ag electrode, affirms the preferential bonding with the O atom of the Cl-doped Ag sample. Theoretical simulations also disclosed that Br-EO adsorbed on the pure Ag plane mainly via non-bonding interactions, but Ag-O bonding contributed to the Br-EO adsorption on Cl-doped Ag.

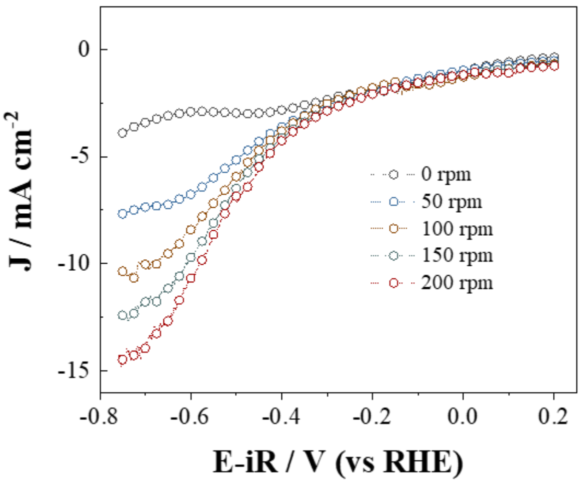

Fig. S31 The LSV curves of the AC-Ag/C electrode were collected in an H-type cell with different stirring speeds.

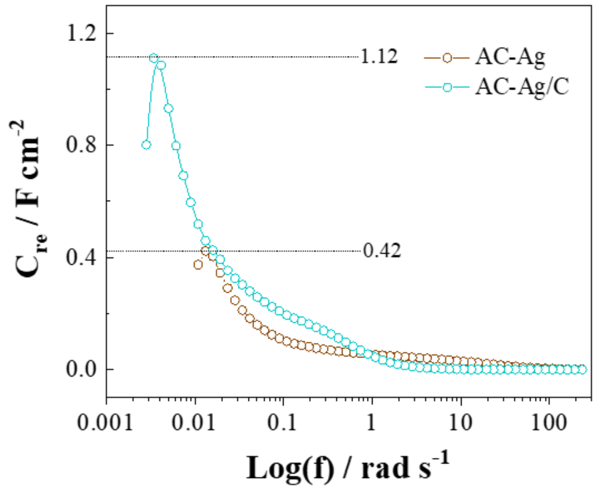

Fig. S32 Evolution of the real part capacitance *vs*. frequency for AC-Ag and AC-Ag/C electrode.

Supplementary Note 5

The capacitances of the real part are 0.42 and 1.12 F cm^{-2} for AC-Ag and AC-Ag/C, respectively. The larger capacitance of AC-Ag/C may stem from the higher specific area of carbon black support, capable of accumulating more charge in the electrochemical double-layer. The significantly higher charges mean an intensive interfacial electric field of AC-Ag/C ($E = V/d$). As known, a larger interfacial electric field can afford a more robust driving force for electron transfer, promoting intermediate adsorption.

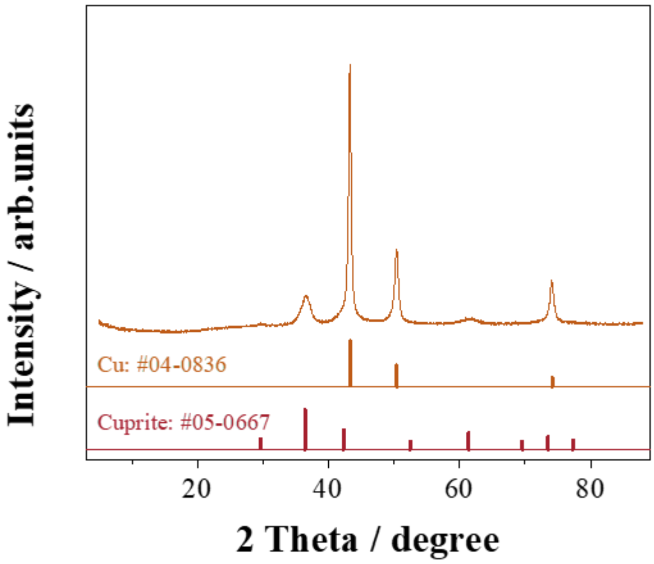

Fig. S33 XRD pattern of Cu/Cu_2O.

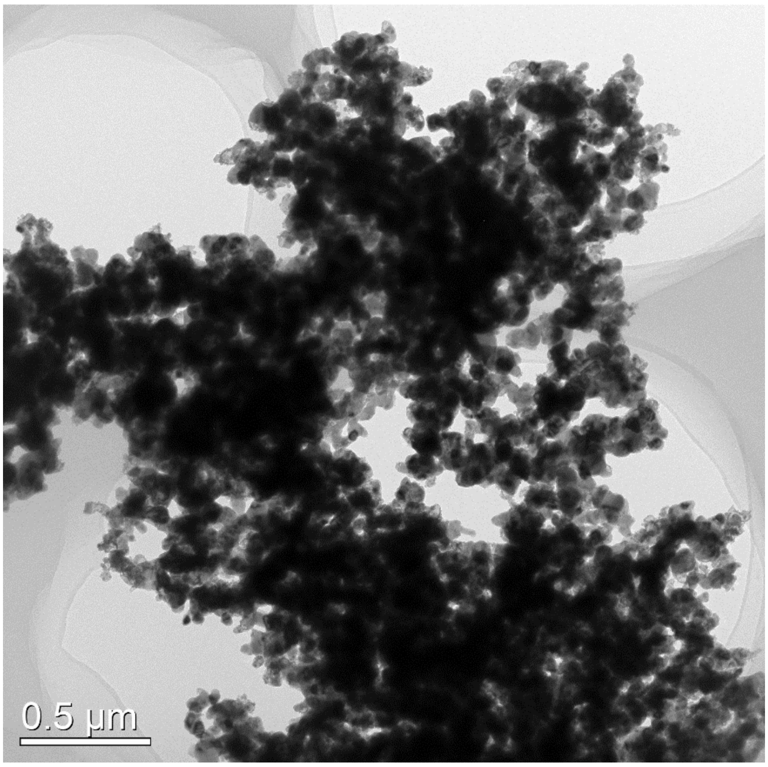

Fig. S34 TEM image of Cu/Cu_2O.

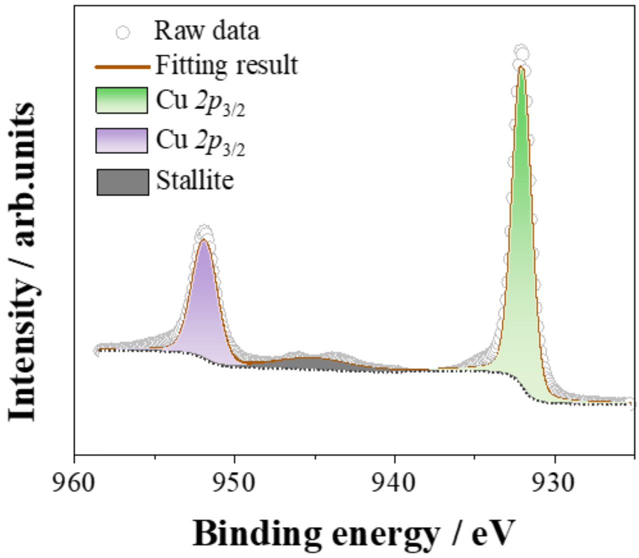

Fig. S35 High-resolution Cu *2p* XPS spectrum of Cu/Cu_2O.

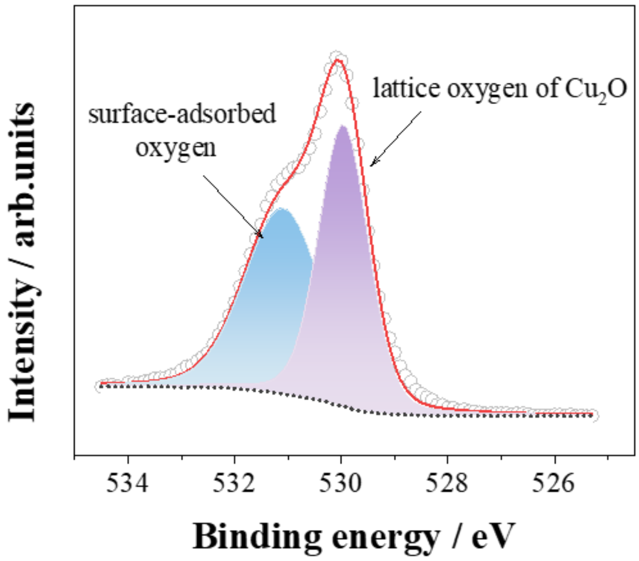

Fig. S36 High solution O *2s* XPS spectrum of Cu/Cu_2O.

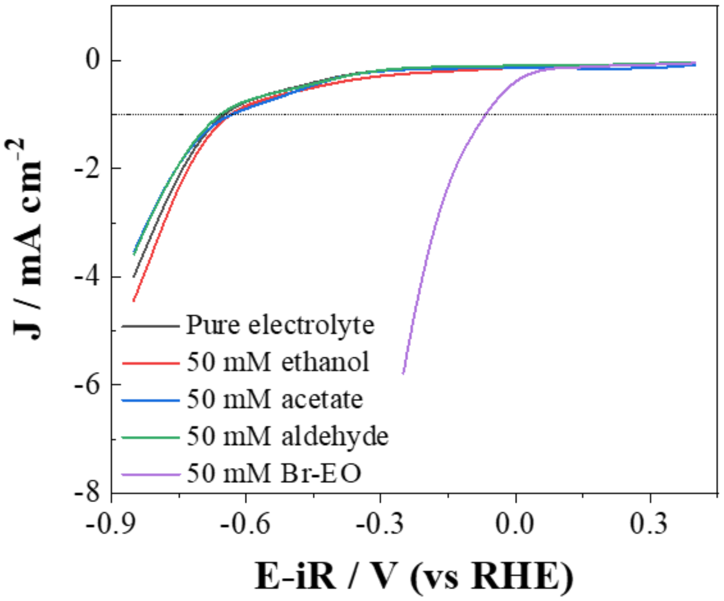

Fig. S37 LSV curves for the reduction of ethanol, acetate, aldehyde, and Br-EO over Ag/C electrode, in 0.5 M KCl electrolyte.

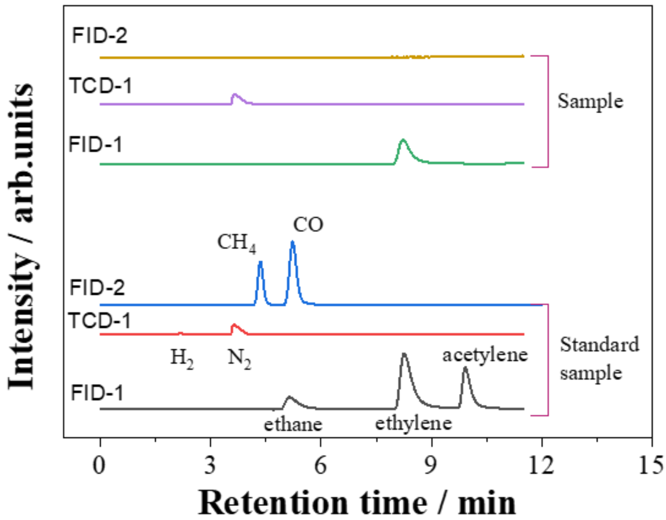

Fig. S38 The GC spectra of the standard sample and the reduction products for remanent electrolyte on Ag/C catalyst. The standard sample used for calibration contains H_2 (103.1 x 10^{-6} mol/mol), CH_4 (10.0 x 10^{-6} mol/mol), C_2H_4 (10.2 x 10^{-6} mol/mol), C_2H_2 (9.97 x 10^{-6} mol/mol), O_2 (0.2000%), CO (9.84 x 10^{-6} mol/mol), C_2H_6 (10.1 x 10^{-6} mol/mol) and N_2.

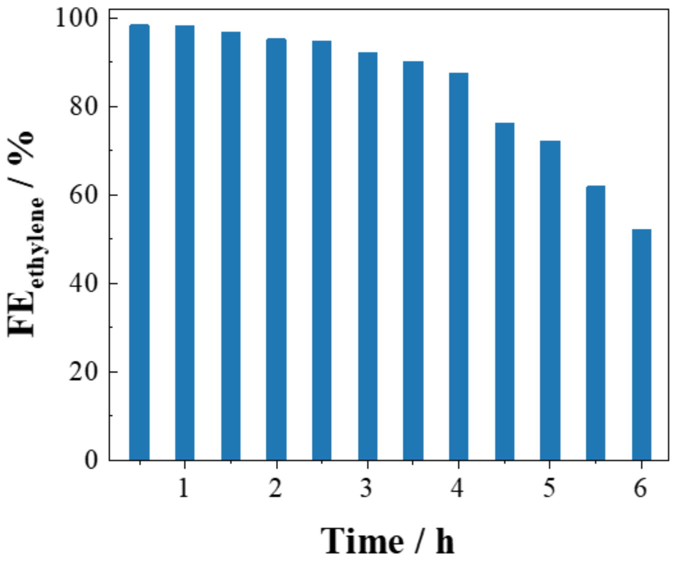

Fig. S39 $FE_{ethylene}$ collected by electrolysis in electrolyte after CO_2 reduction.

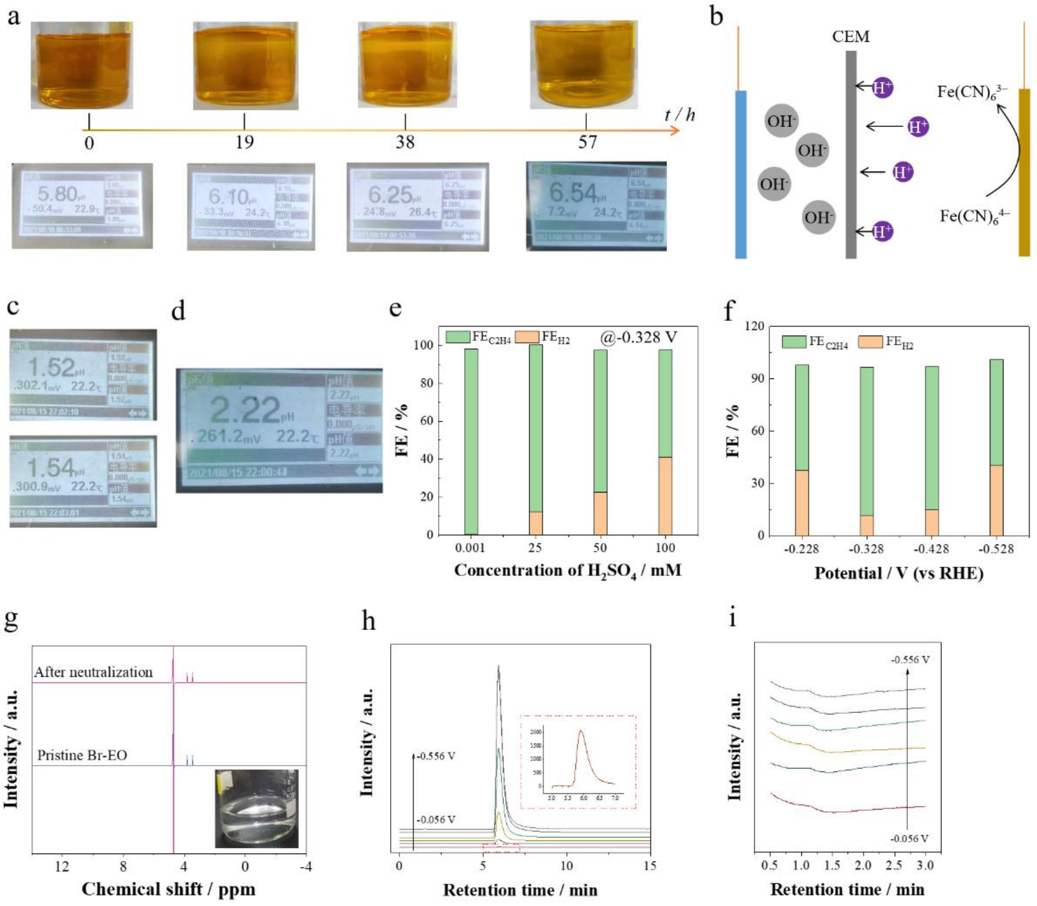

Fig. S40 Analysis for the potential influence of the acidic anolyte. (a) The color and pH of anolyte in the first electrolytic cell after standing for different intervals. (b) Electrolytic system design for evaluating the influence of cation exchange membrane on the pH variation of anolyte. The indication of pH meter for (c) 0.5 M KCl + 25 mM H_2SO_4(upside), 0.5 M KCl + 25 mM H_2SO_4 with CO_2 flow through the cathode chamber for 10 h (downside), and (d) 0.5 M KCl + 25 mM H_2SO_4 + 1 M $KFe(CN)_6$ after electrolysis. (e) FE of H_2 and ethylene for AC-Ag electrode collected in 0.5 M KCl + 50 mM Br-EO with different concentrations of H_2SO_4 at -0.328 V. (f) FE of H_2 and ethylene for AC-Ag electrode collected in 0.5 M KCl + 25 mM H_2SO_4 + 50 mM Br-EO. (g) The ^{1}H NMR of Br-EO in the pristine anolyte and after neutralization with KOH catholyte. Inset is the photo of the anolyte after neutralization. Online GC spectra of (h) FID, (i) TCD detector of the online GC for the Br-EO electroreduction in the neutralized anolyte.

Supplementary Note 6: Discussions about the possible acidic environment of anolyte

We first monitored the pH fluctuations of the anolyte post-electrolysis. A solution with saffron yellow was observed in the anolyte after 2 hours of electrolysis at -200 mA

cm^{-2}, registering a pH of 5.80, indicative of a mild acidic environment (Fig. S40a). After standing for 19 hours, the pH of the anolyte increased to 6.10, subsequently ascending to 6.25 and 6.54 at 38 and 57 hours, respectively. The observed mild acidity, rather than a robust acidic environment, was partially ascribed to the cation exchange membrane (CEM) used in the electrolytic cell. The protons, that are generated in the anolyte, can partially traverse the CEM and interact with hydroxide ions (OH^{-}) present in the catholyte. To substantiate this assertion, we established an electrolysis system featuring a cathodic reaction of CO_2 reduction and anodic oxidation of ferrocyanide ions ($Fe(CN)_6^{4-}$) (Fig. S40b). Notably, the oxidation of $Fe(CN)_6^{4-}$ does not entail the generation or depletion of protons, hence its corresponding influence on the pH variations of the anolyte can be discounted. The aqueous solution of 0.5 M KCl containing 25 mM H_2SO_4 was used as the anolyte (pH = 1.52, as presented in the upside section of Figure S40c). On the cathodic side, CO_2 can react with KOH in the catholyte and generate $KHCO_3$. The resultant HCO_3^{-} may permeate the CEM and further interact with the protons present within the anolyte, potentially modulating its pH. Consequently, we scrutinized the pH of the anolyte following a 10-hour incubation period with continuous CO_2 flow through the cathode chamber. The pH of the anolyte was detected to be 1.54 (downside section of Fig. S40c), almost mirroring that of the pristine electrolyte. This outcome attests to the marginal impact of HCO_3^{-} crossover on the pH dynamics of the anolyte within our experimental framework. Conversely, after the electrolysis of the CO_2 - $Fe(CN)_6^{4-}$ system under -50 mA cm^{-2} for 2 hours, the anolyte was collected, revealing a corresponding pH of 2.22 as indicated by the pH meter (Fig. S40d). This means the electric field-driven proton crossover can significantly buffer the pH reduction of anolyte, thereby preventing the formation of a strong acid environment.

Next, we endeavored to elucidate the impact of an acidic environment on the selectivity of Br-EO electroreduction. As delineated in Fig. S40e, the pH of a 0.5 M KCl aqueous solution was modulated by adding H_2SO_4 across varying concentrations. In a dilute acidic KCl aqueous solution (C_{H2SO4} = 0.001 mM, pH = 5.7), the $FE_{ethylene}$ still reached 98.6%. With the proton concentration increased to 50 mM (equal to the concentration of added Br-EO, pH = 1.59), we also observed a notable ethylene selectivity of 88.5%, and the $FE_{ethylene}$ remaining above 50.0% even upon introducing 0.1 M H_2SO_4. In the electrolyte comprising 0.5 M KCl + 25 mM H_2SO_4 + 50 mM Br-EO, ethylene emerges as the principal product across the potential window ranging from -0.228 to -0.528 (Fig. S40f). These findings underscore the feasibility of achieving high-purity ethylene production within a mild acidic solution.

Finally, the alkaline catholyte is gradually added into the anolyte, to remove the residue Br_2 and neutralize the formed acid, transforming the solution from saffron yellow to colorless, as depicted in the inset of Fig. S40g. Notably, the gradually added alkaline catholyte did not induce any changes in the Br-EO in the anolyte, as confirmed by the ^1H NMR (Fig. S40g). Subsequently, the electrolysis of this mixed solution by AC-Ag electrode gave the exclusive ethylene peak in the online GC, with the $FE_{ethylene}$ exceeding 95.0%, with no identifiable peak for H_2 (retention time of 2.25 min) observed (Fig. S40h-S40i). These results further support that the neutralization of anolyte by the catholyte of the first electrolytic cell can effectively remove the potential influence of a weak acid environment.

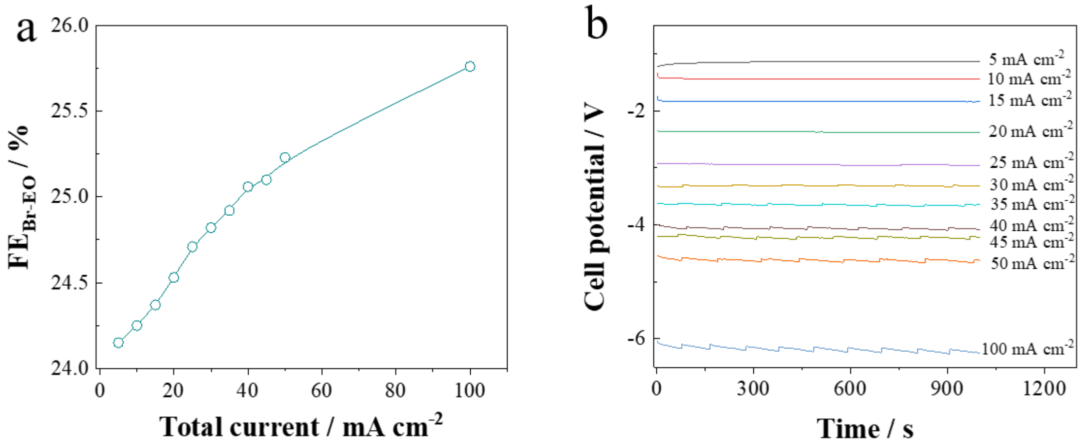

Fig. S41 Electrochemical performance of Br-EO reduction. (a) The required FE_{Br-EO} of the indirect route for achieving the comparable energy to that of the direct route at different total currents. (b) The cell potentials for acquiring different current densities that collected by galvanostatic analysis.

Supplementary Note 7: Energy consumption for direct CO_2-to-ethylene conversion.

The specific energy for electrochemical C_2H_4 production can be calculated via the following equation.[1]

$$W_E = \frac{4.0\ \text{V}}{1.15\ \text{V} \times 0.75} \times 47.5 \frac{\text{GJ}}{\text{tonne C2H4}}$$

$$= 220.3\ \text{GJ/tonne } C_2H_4$$

Considering the energy input for CO_2 and H_2O removal, and the ethylene separation, the total energy consumption (W_{direct}) is 564.3 (220.3 + 279 + 55+ 10) GJ/tonne ethylene.

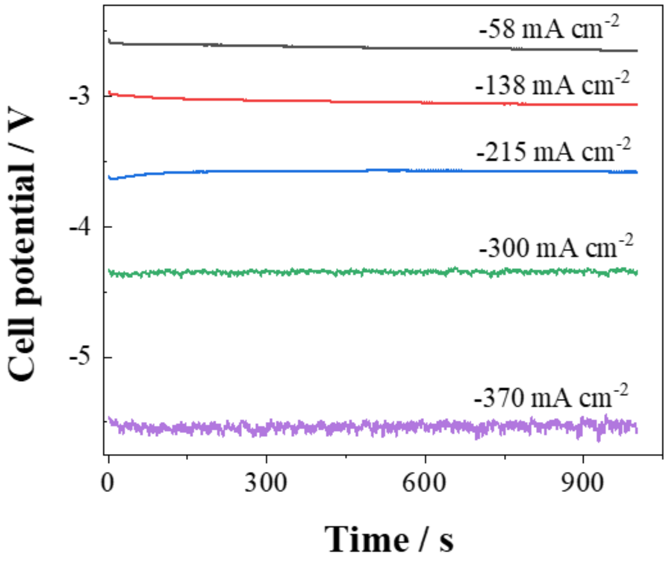

Fig. S42 Cell potentials for CO_2-to-Br-EO conversion under different current densities.

Supplementary Note 8: Details for the total electrical work calculation for our indirect route.

$$m_{\text{Br-EO}}=\frac{I\times t}{F\times N}\times \text{FE}_{\text{Br-EO}}\times M_{\text{Br-EO}}$$

If t=1 s, then

$$m_{\text{Br-EO}}/\text{s}=\frac{I}{F\times N}\times \text{FE}_{\text{Br-EO}}\times M_{\text{Br-EO}}$$

The quantity of Br-EO required to produce 1 tonne of ethylene can be calculated as follows:

$$m'_{\text{Br-EO}}=\frac{10^6\ g}{M_{\text{ethylene}}}\times M_{\text{Br-EO}}$$

The time (t') to obtain this Br-EO is given by

$$t'=\frac{m'_{\text{Br-EO}}}{m_{\text{Br-EO}}/\text{s}}$$

Then the corresponding electrical work for CO_2-to-Br-EO conversion can be determined as

$$W_1=U\times I\times t'$$

$$=U\times I\times \frac{10^6\times M_{\text{Br-EO}}}{M_{\text{ethylene}}}\times \frac{F\times N}{I\times \text{FE}_{\text{Br-EO}}\times \text{M}_{\text{Br-EO}}}$$

$$=U\times \frac{10^6}{M_{\text{ethylene}}}\times \frac{F\times N}{\text{FE}_{\text{Br-EO}}}$$

$$=U\times \frac{10^6}{28}\times \frac{96485\times 10}{\text{FE}_{\text{Br-EO}}}$$

$$=\frac{U}{FE_{Br\text{-}EO}}\times 34.46\ \text{GJ/tonne}$$

$$=\frac{3.59}{0.461}\times 34.46\frac{\text{GJ}}{\text{tonne}}$$

$$= 268.35\ \text{GJ/tonne}$$

Similarly, as for the reduction of Br-EO to ethylene, the corresponding electrical work can be obtained as follows:

$$W_2=U\times I\times\frac{10^6\times F\times N}{I\times M_{\text{ethylene}}\times \text{FE}_{\text{ethylene}}}$$

Considering the exclusive conversion of Br-EO to ethylene over our Ag-based electrocatalysts,

$$W_2=U\times\frac{10^6\times F\times N}{M_{\text{ethylene}}}$$

$$=U\times\frac{10^6\times 96485\times 2}{28}$$

$$= U\times 6.89\ \text{GJ/tonne}$$

Using the cell potential at the current density of -100 mA cm^{-2}, namely 6.19 V,

$$W_2=6.19 \text{ x } 6.89\frac{\text{GJ}}{\text{tonne}}$$

$$= 42.65\ \text{GJ/tonne}$$

The total energy consumption W = W_1 + W_2 = (268.35+42.65) GJ/tonne = 310.99 GJ/tonne.

Supplementary Note 9: Discussions about the possible corrosion problems of Br_2-derived species.

Concerning potential remedies, firstly, insights can be gleaned from established practices in the bromine industry and chlor-alkali industry, including their storage technologies and secure production protocols. For example, the utilization of a sunblock strategy for the anode chamber is conceivable. Given our operations at room temperature and atmospheric pressure, glass-lined vessels can endure the rigors of Br_2-involved testing, while vessels with ceramic linings also prove viable. The oxidative corrosion potency of Cl_2 surpasses that of Br_2; hence, the design of electrolysis cells (comprising structure, separator, and texture) and electrode design can provide valuable insights for our Br_2-involved indirect pathway. Notably, stable Pt and IrO_2/TiO_2 electrodes employed in the chlor-alkali industry can seamlessly integrate into our system. Furthermore, safety specifications established for the chlor-alkali industry offer

guidance in mitigating safety concerns within our system.

Secondly, as described in our manuscript, the electrolyte post-CO_2-to-Br-EO conversion was collected, which is further used as the electrolyte for ethylene formation via Br-EO electroreduction. To prevent excessive accumulation, periodic transfer of electrolytes from the CO_2 electrochemical cell to the Br-EO electroreduction system is conceivable. Importantly, if the Br-EO formation rate proves sufficiently high to sustain subsequent electroreduction, the adoption of a continuous flow reactor becomes tenable. This approach ensures a perpetual influx of fresh electrolytes, thereby preventing an excessive concentration of Br_2 species and concurrently mitigating corrosion and safety concerns. In this flow cell, worth noting is the potential circulation of the electrolyte after Br-EO reduction, enriched with regenerated Br^-, for anodic oxidation in the CO_2-to-Br-EO conversion system.

Table S1. The FE and the purity of ethylene for Br-EO reduction in electrolytes with different concentrations.

Potential / V (vs RHE)	Concentration of Br-EO / mM	$FE_{ethylene}$ / %	Purity / (m) %
-0.08	50	95.9	99.39
-0.18	50	96.0	99.39
-0.28	50	97.2	99.49
-0.38	50	98.0	99.55
-0.48	50	97.2	99.49
-0.58	50	97.6	99.52
-0.08	25	98.3	99.57
-0.18	25	100.1	99.69
-0.28	25	99.7	99.67
-0.38	25	101.2	99.69
-0.48	25	101.3	99.69
-0.58	25	96.8	99.46
-0.08	10	98.1	99.55
-0.18	10	95.7	99.37
-0.28	10	97.5	99.51
-0.38	10	99.3	99.64
-0.48	10	98.2	99.56
-0.58	10	97.7	99.52
-0.08	5	95.3	99.34
-0.18	5	97.6	9952
-0.28	5	98.7	99.60
-0.38	5	97.0	99.47
-0.48	5	99.4	99.65
-0.58	5	96.7	99.45
-0.08	2	61.5	95.42
-0.18	2	96.5	99.43
-0.28	2	96.4	99.43
-0.38	2	92.6	99.22
-0.48	2	84.8	98.43
-0.58	2	71.8	96.96

Supplementary Note 10: It can be found that the low $FE_{ethylene}$ was observed in electrolytes with the Br-EO concentration of 2 mM, under the potentials of -0.08, -0.48, and -0.58 V. The relatively diminished FE at -0.08 V (61.5%) is likely attributed to the influence of the double-layer charging effect, considering the exceedingly low current density at this potential (-0.25 mA cm^{-2}). In contrast, higher current densities were recorded for electrolytes containing 5 mM (-0.85 mA cm^{-2}), 10 mM (-1.35 mA cm^{-2}), 25 mM (-1.6 mA cm^{-2}), and 50 mM (-3.0 mA cm^{-2}) Br-EO. This can mediate the impact of capacitance current, thereby giving the $FE_{ethylene}$ higher than 95.0%. While for the low $FE_{ethylene}$ at -0.48 and -0.58 V, it can be reasonably ascribed to the limitations imposed by the mass transfer behavior of Br-EO under the low concentration electrolyte, because such low selectivity was not observed in the electrolytes containing more Br-EO. With the increasing concentration of Br-EO, the mass transfer-induced limiting currents progressively dissipated, culminating in a complete disappearance of these limitations at 50 mM Br-EO.

Table S2

Catalyst	Purity / (mol)%	Potential / V (vs RHE)	Reference
Cu_3N nanocubes	0.33	-1.6	[2]
Cu with electro-dimerized arylpyridinium	0.91	-0.83	[3]
I modified Cu	0.62	-1.1	[4]
4H Cu	0.13	-1.1	[5]
Anodized Cu	0.06	-1.05	[6]
$Cu_3(HITP)_2$-KB	1.85	-1.35	[7]
B-doped CuO nanobundles	0.06	-1.1	[8]
Branched copper oxides	0.04	-1.05	[9]
Cu-NiNC	<0.05	-1.1	[10]
CuO/Al_2CuO_4	<0.01	-1	[11]
F-doped Cu	3.2	-0.75	[12]
Cu in local alkaline interface	0.86	-0.9	[13]
Cu-polyamine	5.5	-0.97	[14]
Cu with nanoscaled defects	0.29	-1.2	[15]
Cu/CuxSy	0.56	-1.35	[16]
Cu in high concentrated KOH	2.2	-0.55	[17]
Cu/Ionic liquids	0.13	-1.49	[18]
Cu/Cu_2O	1.04	-0.81	[19]
$Cu\text{-}SiO_x$	0.33	N/A	[20]
Cu_2O	0.06	-1.1	[21]
Au-Cu MOF	0.05	-1.2	[22]
Cu_3 cluster	0.44	-0.7	[23]
Cu–Au/Ag	0.57	-1.2	[24]
MgAl-LDH/Cu	0.93	N/A	[25]
Cu/Al_2O_3	2.22	-1.1	[26]
Cu/CeO_2	0.35	-0.7	[27]
Cu/bifunctional ionomers	1.0	-1.4	[28]
Cu_2O	0.18	-1.2	[29]
MOF derived Cu/Cu_2O	0.03	-1.03	[30]
Cu	7.5	-0.55	[31]
Cu/PTFE	12.8	N/A	[32]
Cationic group functional Cu	37.0	N/A	[33]
Cu/ionomer	11.8	N/A	[34]
Indirect reduction route	94.1 (mean value of 4 hours electrolysis)	-0.48	this work

The following equations have been accepted to ascertain the ethylene purity on a molar basis.

$$P_{\text{ethylene}} = \frac{flux_{\text{ethylene}}}{flux_{\text{total}}} \times 100$$

$$flux_{\text{ethylene}} = \frac{j_{\text{ethylene}}}{N_{\text{ethylene}} \times F}$$

Where flux is the flux of gaseous chemicals, j is the current density, N is the number of electrons transferred, and F is Faraday's constant.

Supplementary Note 11: The influence of vapor in the product stream on the ethylene purity.

We performed a quantitative analysis of the water content in the product stream using the apparatus shown in Fig. S43a. Gas from the cathode chamber initially passed through a flask containing 5.0 g of ultra-dry N,N-dimethylacetamide (DME), known for its effective water vapor adsorption. To prevent atmospheric moisture from affecting results, a tail-gas dryer was attached to the flask's opposite end. Water content in the DME was subsequently measured using a Karl-Fischer titrator to quantify water captured from the exhaust gas of the cathode chamber.

As presented in Fig. S43b, the initial water content of the pristine DME is 254.3±31.6 ppm. After 48 hours of electrolysis at a constant current of -30 mA cm^{-2}, with periodic addition of Br-EO to the catholyte, the detected water content in DME increased to 1223±142 ppm. Therefore, the net increase in water content after electrolysis is 968.7 ppm. Considering the near-complete conversion of ethylene, the water content in the electrolysis product can be calculated as follows:

$$H_2O\ wt\% = \frac{968.7\times10^{-6}\times5.0}{\frac{0.03\times48\times3600}{96485}\times28}$$

$$=0.322wt\%$$

Therefore, the mass fraction of H_2O in the product stream is 0.322% when the current density is -30 mA cn^{-2}. Based on this data, we corrected the purity of ethylene reported in our manuscript.

$$P_{ethylene}(m\%) = \frac{FE\times M_{ethylene}}{\frac{FE_{ethylene}\times M_{ethylene}+FE_{H2}\times M_{H2}}{100-0.322}}$$

Using this equation, the average ethylene purity during the continuous electrolysis over 6 hours in the integrated indirect route was corrected to 98.00%, which is 0.31 wt% lower than previously reported without accounting for water vapor in the product stream. This difference is likely overestimated due to the current density gradually decreasing with the consumption of Br-EO, resulting in a lower water content in the gaseous product than calculated under constant current conditions.

Due to the low water content in the product stream, a simple water absorber, connected to the outlet of the cathode chamber, can efficiently remove the water. Therefore, we placed an absorption trap, containing color-silica gel (30 g), between the cathode chamber and the ultra-dry DME (Fig. S43c). After continuous electrolysis of 48 hours at -30 mA cm^{-2}, the water content was 211.7±11.5 ppm (Fig. S43d), close to that of pristine ultra-dry DME. Thus, the absorption trap can nearly remove all the water in the product stream under our testing scenario.

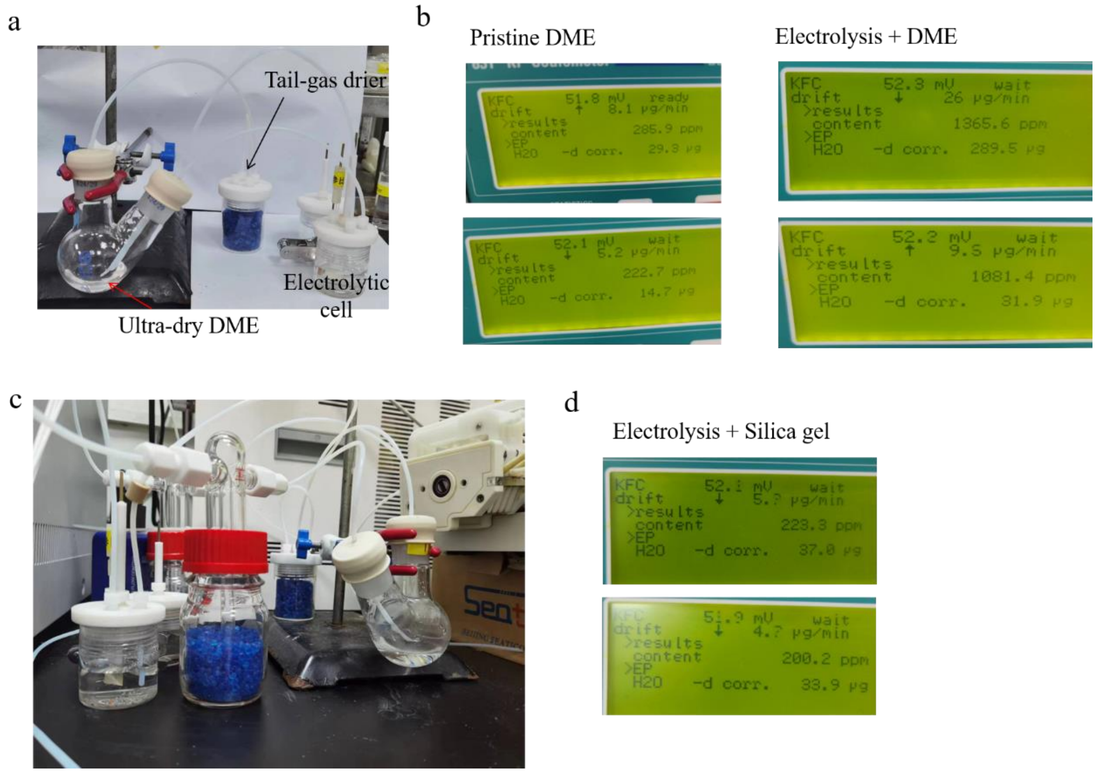

Fig. S43 (a) Setup for quantitative analysis of water content carried out by product stream. (b) The content of water in DME for pristine ultra-dry DME, and Electrolysis + DME. (c) Setup for adsorbing water carried out by product stream. (d) The reading in Karl-Fischer titrator for Electrolysis + Silica gel.

Supplementary references

[1] T. Alerte, J. P. Edwards, C. M. Gabardo, C. P. O'Brien, A. Gaona, J. Wicks, A. Obradovi, A. Sarkar, S. A. Jaffer, H. L. MacLean, D. Sinton, E. H. Sargent, *ACS Energy Lett.* **2021**, 6, 4405.

[2] Z. Y. Yin, C. Yu, Z. L. Zhao, X. F. Guo, M. Q. Shen, N. Li, M. Muzzio, J. R. Li, H. Liu, H. H. Lin, J. Yin, G. Lu, D. Su, S. H. Sun, *Nano Lett.* **2019**, 19, 8658.

[3] F. W. Li, A. Thevenon, A. Rosas-Hernandez, Z. Y. Wang, Y. L. Li, C. M. Gabardo, A. Ozden, C. T. Dinh, J. Li, Y. H. Wang, J. P. Edwards, Y. Xu, C. McCallum, L. Z. Tao, Z. Q. Liang, M. C. Luo, X. Wang, H. H. Li, C. P. O'Brien, C. S. Tan, D. H. Nam, R. Quintero-Bermudez, T. T. Zhuang, Y. G. C. Li, Z. J. Han, R. D. Britt, D. Sinton, T. Agapie, J. C. Peters, E. H. Sargent, *Nature* **2020**, 577, 509.

[4] D. F. Gao, I. Sinev, F. Scholten, R. M. Aran-Ais, N. J. Divins, K. Kvashnina, J. Timoshenko, B. Roldan Cuenya, *Angew. Chem. Int. Ed.* **2019**, 58, 17047.

[5] Y. Chen, Z. X. Fan, J. Wang, C. Y. Ling, W. X. Niu, Z. Q. Huang, G. G. Liu, B. Chen, Z. C. Lai, X. Z. Liu, B. Li, Y. Zong, L. Gu, J. L. Wang, X. Wang, H. Zhang, *J. Am. Chem. Soc.* **2020**, 142, 12760.

[6] S. Y. Lee, H. Jung, N. K. Kim, H. S. Oh, B. K. Min, Y. J. Hwang, *J. Am. Chem. Soc.* **2018**, 140, 8681.

[7] H. Sun, L. Chen, L. Xiong, K. Feng, Y. Chen, X. Zhang, X. Yuan, B. Yang, Z. Deng, Y. Liu, M. H. Rummeli, J. Zhong, Y. Jiao, Y. Peng, *Nature communications* **2021**, 12, 6823.

[8] Q. Wan, J. L. Zhang, B. X. Zhang, D. X. Tan, L. Yao, L. R. Zheng, F. Y. Zhang, L. F. Liu, X. Y. Cheng, B. X. Han, *Green Chem.* **2020**, 22, 2750.

[9] J. Kim, W. Choi, J. W. Park, C. Kim, M. Kim, H. Song, *J. Am. Chem. Soc.* **2019**, 141, 6986.

[10] D. L. Meng, M. D. Zhang, D. H. Si, M. J. Mao, Y. Hou, Y. B. Huang, R. Cao, *Angew. Chem. Int. Ed.* **2021**, 60, 25485.

[11] S. Sultan, H. Lee, S. Park, M. M. Kim, A. Yoon, H. Choi, T. H. Kong, Y. J. Koe, H. S. Oh, Z. Lee, H. Kim, W. Kim, Y. Kwon, *Energy. Environ. Sci.* **2022**, 15, 2397.

[12] W. C. Ma, S. J. Xie, T. T. Liu, Q. Y. Fan, J. Y. Ye, F. F. Sun, Z. Jiang, Q. H. Zhang, J. Cheng, Y. Wang, *Nat. Catal.* **2020**, 3, 478.

[13] Z. Wang, Y. Li, X. Zhao, S. Chen, Q. Nian, X. Luo, J. Fan, D. Ruan, B. Q. Xiong, X. Ren, *J Am Chem Soc* **2023**, doi: 10.1021/jacs.2c13384.

[14] X. Y. Chen, J. F. Chen, N. M. Alghoraibi, D. A. Henckel, R. X. Zhang, U. O. Nwabara, K. E. Madsen, P. J. A. Kenis, S. C. Zimmerman, A. A. Gewirth, *Nat. Catal.* **2021**, 4, 20.

[15] B. X. Zhang, J. L. Zhang, M. L. Hua, Q. Wan, Z. Z. Su, X. N. Tan, L. F. Liu, F. Y. Zhang, G. Chen, D. X. Tan, X. Y. Cheng, B. X. Han, L. R. Zheng, G. Mo, *J. Am. Chem. Soc.* **2020**, 142, 13606.

[16] C. F. Wen, M. Zhou, P. F. Liu, Y. W. Liu, X. F. Wu, F. X. Mao, S. Dai, B. B. Xu, X. L. Wang, Z. Jiang, P. Hu, S. Yang, H. F. Wang, H. G. Yang, *Angew. Chem. Int. Ed.* **2022**, 61, e202111700.

[17] C. T. Dinh, T. Burdyny, M. G. Kibria, A. Seifitokaldani, C. M. Gabardo, F. P. G. de Arquer, A. Kiani, J. P. Edwards, P. De Luna, O. S. Bushuyev, C. Q. Zou, R. Quintero-Bermudez, Y. J. Pang, D. Sinton, E. H. Sargent, *Science* **2018**, 360, 783.

[18] Y. F. Sha, J. L. Zhang, X. Y. Cheng, M. Z. Xu, Z. Z. Su, Y. Y. Wang, J. Y. Hu, B. X. Han, L. R. Zheng, *Angew. Chem. Int. Ed.* **2022**, 61, e202200039.

[19] W. Liu, P. B. Zhai, A. W. Li, B. Wei, K. P. Si, Y. Wei, X. G. Wang, G. D. Zhu, Q. Chen, X. K. Gu, R. F. Zhang, W. Zhou, Y. J. Gong, *Nat. Commun.* **2022**, 13, 1877.

[20] J. Li, A. Ozden, M. Y. Wan, Y. F. Hu, F. W. Li, Y. H. Wang, R. R. Zamani, D. Ren, Z. Y. Wang, Y. Xu, D. H. Nam, J. Wicks, B. Chen, X. Wang, M. C. Luo, M. Graetzel, F. L. Che, E. H. Sargent, D. Sinton, *Nat. Commun.* **2021**, 12, 2808.
[21] Y. G. Gao, Q. Wu, X. Z. Liang, Z. Y. Wang, Z. K. Zheng, P. Wang, Y. Y. Liu, Y. Dai, M. H. Whangbo, B. B. Huang, *Adv. Sci.* **2020**, 7, 1902820.
[22] X. L. Xie, X. Zhang, M. Xie, L. K. Xiong, H. Sun, Y. T. Lu, Q. Q. Mu, M. H. Rummeli, J. B. Xu, S. Li, J. Zhong, Z. Deng, B. Y. Ma, T. Cheng, W. A. Goddard, Y. Peng, *Nat. Commun.* **2022**, 13, 63.
[23] Y. F. Lu, L. Z. Dong, J. Liu, R. X. Yang, J. J. Liu, Y. Zhang, L. Zhang, Y. R. Wang, S. L. Li, Y. Q. Lan, *Angew. Chem. Int. Ed.* **2021**, 60, 26210.
[24] L. K. Xiong, X. Zhang, H. Yuan, J. Wang, X. Z. Yuan, Y. B. Lian, H. D. Jin, H. Sun, Z. Deng, D. Wang, J. P. Hu, H. M. Hu, J. Choi, J. Li, Y. F. Chen, J. Zhong, J. Guo, M. H. Rummerli, L. Xu, Y. Peng, *Angew. Chem. Int. Ed.* **2021**, 60, 2508.
[25] Y. N. Xu, W. Li, H. Q. Fu, X. Y. Zhang, J. Y. Zhao, X. Wu, H. Y. Yuan, M. Zhu, S. Dai, P. F. Liu, H. Yang, *Angew. Chem. Int. Ed.* **2023**, e202217296.
[26] H. Li, P. P. Yu, R. B. Lei, F. P. Yang, P. Wen, X. Ma, G. S. Zeng, J. H. Guo, F. M. Toma, Y. J. Qiu, S. M. Geyer, X. W. Wang, T. Cheng, W. S. Drisdell, *Angew. Chem. Int. Ed.* **2021**, 60, 24838.
[27] D. X. Tan, B. Wulan, X. Y. Cao, J. T. Zhang, *Nano Energy* **2021**, 89, 106460.
[28] W. Z. Li, Z. L. Yin, Z. Y. Gao, G. W. Wang, Z. Li, F. Y. Wei, X. Wei, H. Q. Peng, X. T. Hu, L. Xiao, J. T. Lu, L. Zhuang, *Nat. Energy* **2022**, 7, 835.
[29] H. Q. Luo, B. Li, J. G. Ma, P. Cheng, *Angew. Chem. Int. Ed.* **2022**, 61, e202116736.
[30] C. Liu, X. D. Zhang, J. M. Huang, M. X. Guan, M. Xu, Z. Y. Gu, *Acs Catal.* **2022**, 12, 15230.
[31] C. P. O’Brien, R. K. Miao, S. Liu, Y. Xu, G. Lee, A. Robb, J. E. Huang, K. Xie, K. Bertens, C. M. Gabardo, J. P. Edwards, C.-T. Dinh, E. H. Sargent, D. Sinton, *Acs Energy Lett* **2021**, 6, 2952.
[32] X. Zi, Y. J. Zhou, L. Zhu, Q. Chen, Y. Tan, X. Q. Wang, M. Sayed, E. Pensa, R. A. Geioushy, K. Liu, J. W. Fu, E. Cortes, M. Liu, *Angew. Chem. Int. Ed.* **2023**, 62, e202309351.
[33] M. Y. Fan, J. E. Huang, R. K. Miao, Y. Mao, P. F. Ou, F. Li, X. Y. Li, Y. F. Cao, Z. S. Zhang, J. Q. Zhang, Y. Yan, A. Ozden, W. Y. Ni, Y. Wang, Y. Zhao, Z. Chen, B. Khatir, C. P. O'Brien, Y. Xu, Y. C. Xiao, G. I. N. Waterhouse, K. Golovin, Z. Y. Wang, E. H. Sargent, D. Sinton, *Nat. Catal.* **2023**, 6, 763.
[34] Y. Zhao, L. Hao, A. Ozden, S. Liu, R. K. Miao, P. Ou, T. Alkayyali, S. Zhang, J. Ning, Y. Liang, Y. Xu, M. Fan, Y. Chen, J. E. Huang, K. Xie, J. Zhang, C. P. O’Brien, F. Li, E. H. Sargent, D. Sinton, *Nature Synthesis* **2023**, 2, 403.
